# A Novel Golgi Retention Signal RPWS for Tumor Suppressor UBIAD1

**DOI:** 10.1371/journal.pone.0072015

**Published:** 2013-08-19

**Authors:** Xian Wang, Dangfeng Wang, Pan Jing, Yuangan Wu, Yanzhi Xia, Maorong Chen, Ling Hong

**Affiliations:** 1 Department of Genetics and Developmental Biology, College of Life Science and Technology,Huazhong University of Science and Technology, Wuhan, Hubei, China; 2 Department of Biophysics, College of Life Science and Technology,Huazhong University of Science and Technology, Wuhan, Hubei, China; Northwestern University Feinberg School of Medicine, United States of America

## Abstract

UBIAD1 plays critical roles in physiology including vitamin K and CoQ10 biosynthesis as well as pathophysiology including dyslipimedia-induced SCD (Schnyder’s corneal dystrophy), Parkinson’s disease, cardiovascular disease and bladder carcinoma. Since the subcellular localization of UBIAD1 varies in different cell types, characterization of the exact subcellular localization of UBIAD1 in specific human disease is vital for understanding its molecular mechanism. As UBIAD1 suppresses bladder carcinoma, we studied its subcellular localization in human bladder carcinoma cell line T24. Since fluorescent images of UBIAD1-EGFP in T24, human prostate cancer cell line PC-3, human embryonic kidney cell line HEK293 and human hepatocyte cell line L02 are similar, these four cell lines were used for present study. Using a combination of fluorescent microscopy and immunohistochemistry, it was found that UBIAD1 localized on the Golgi and endoplasmic reticulum (ER), but not on the plasma membrane, of T24 and HEK293 cells. Using scanning electron microscopy and western blot analysis, we found that UBIAD1 is enriched in the Golgi fraction extracted from the L02 cells, verifying the Golgi localization of UBAID1. Site-directed mutagenesis showed that the RPWS motif, which forms an Arginine finger on the UBIAD1 N terminus, serves as the Golgi retention signal. With both cycloheximide and brefeldin A inhibition assays, it was shown that UBIAD1 may be transported from the endoplasmic reticulum (ER) to the Golgi by a COPII-mediated mechanism. Based upon flow cytometry analysis, it is shown that mutation of the RPWS motif reduced the UBIAD1-induced apoptosis of T24 cells, indicating that the proper Golgi localization of UBIAD1 influences its tumor suppressant activity. This study paves the way for further understanding the molecular mechanism of UBIAD1 in human diseases.

## Introduction

Bladder carcinoma is one of the most common causes of cancer worldwide. In 2013, there will be an estimated 72,570 new cases of bladder cancer and 15,210 bladder cancer related deaths in US alone [1]. *Ubiad1*, also known as *Tere1* (transitional epithelial response gene), was first cloned as a tumor suppressor for human bladder carcinoma [2], [3]. *Ubiad1* (UbiA prenyltransferase domain containing 1) encodes a class of UbiA prenyltransferase involved in SCD (Schnyder’s corneal dystrophy), a rare dominant genetic eye disease [4], [5], [6]. The main phenotype of SCD is dyslipidemia which leads to the local accumulation of cholesterol, causing progressive corneal pacification [7], [8]. Furthermore, UBIAD1 protein has been shown to physically interact with apolipoprotein E and can lower the intracellular cholesterol level in HEK293 cells [9], [10].

In addition to its roles as a tumor suppressor and as a modulator for intracellular cholesterol, UBIAD1 has been shown to be the first enzyme responsible for human vitamin K biosynthesis [11]. UBIAD1 encodes a novel human menaquinone-4 biosynthetic enzyme, converting the vitamin K derivatives to MK-4. The prenyltransferase activity of UBIAD1 is responsible for cleaving the side chain from vitamin K derivatives and substituting it with a geranylgeranyl side chain. Vos et al. reported that *Drosophila ubiad1/heix* is a modifier of *pink1*, a gene mutated in Parkinson’s disease that affects mitochondrial function [12]. Recently, Mugoni et al. showed [13] that UBIAD1 is a prenyltransferase required for CoQ10 biosynthesis in Golgi membranes, protecting cardiovascular system from reactive oxygen species (ROS) by regulating eNOS activity.

Our previous report has shown that UBIAD1 is a negative regulator of the Ras-MAPK signaling pathway [14]. Knockdown of UBIAD1 expression with siRNA directly activates the Ras-MAPK signal transduction pathway, resulting in upregulation of hTERT transcription and ultimately cell proliferation. In our study of the *Drosophila heix* gene, which encodes an ortholog of human UBIAD1, we observed a correlation between the malignant blood tumor phenotype and an increased number of *Drosophila* blood cells carrying mutant nonfunctional *heix*. This is consistent with the role of UBIAD1 being a bona fide tumor suppressor [2], [14].

Despite the aforementioned reports about the various biological functions of UBIAD1, its subcellular localization remains to be elucidated. Studies on the subcellular localization of wild type and mutant human UBIAD1 showed that neither the wild type nor the mutant UBIAD1 colocalizes with an enzyme marker (protein disulfide isomerase) on endoplasmic reticulum inside cultured human keratocytes [15]. On the other hand, N102S mutant UBIAD1 did colocalize with a mitochondrial marker (OXPHOS complex I, NADH dehydrogenase) in keratocytes derived from the SCD family. In addition, Nakagawa et al. showed that UBIAD1-GFP colocalized with the endoplasmic reticulum marker (ER-tracker Red), but not with the Golgi marker (BODIPY-TR ceramide) in human osteoblast-like MG-63 cells [11]. *Drosophila* UBIAD1/Heix expressed in S2 cells localized to the mitochondria, as in human keratocytes [12], [15]. Recently, Mugoni et al. showed [13] that UBIAD1 is localized in Golgi membranes in human endothelial cells.

During the biogenesis of proteins in cells, the Golgi apparatus is the main venue for protein sorting. The newly synthesized secretory proteins or proteins for the endomembrane system of secretory pathways are transported via anterograde trafficking pathways from the endoplasmic reticulum (ER) to the Golgi. Here, these proteins are further modified at post-translational level and delivered to different intracellular or extracellular locations. On the other hand, proteins can also be transported from the Golgi to the ER via retrograde trafficking pathways. Proteins are retained in the Golgi by various different mechanisms such as recognition and binding of the proteins by COPII complexes [16], [17], [18], [19], [20]. In the Golgi, many signal transduction pathways are intimately involved in the protein retention and trafficking process [21]. Deciphering the molecular mechanism of protein retention and trafficking in the Golgi will greatly benefit our understanding of the intracellular trafficking process as well as compartmentalized signaling.

The discrepancy between different reports of UBIAD1 subcellular localization compelled us to further investigate this issue in bladder carcinoma cells as UBIAD1 has been shown to be a tumor suppressor for bladder carcinoma [2], [10], [14]. Using a combination of biochemical and cellular approaches, we found in the present study that UBIAD1 accumulates on the Golgi in human bladder carcinoma cell line T24. The Golgi retention signal of UBIAD1 is a novel protein motif, RPWS, which influences the tumor suppressing activity of UBIAD1.

## Materials and Methods

### Plasmid Constructs

The plasmid pOTB7-UBIAD1 was purchased from Open Biosystems (USA). The enhanced green fluorescent protein (EGFP) vector plasmid EGFP-N1 and Casper 3-BG, a mammalian expression vector (TagBFP), were obtained from Invitrogen (USA). Plasmid DsRed2, a mammalian expression vector that encodes a fusion protein consisting of Discosoma sp. red fluorescent protein (DsRed2), was obtained from Clontech (USA). For plasmid ER-RFP, an endoplasmic reticulum (ER) targeting sequence of calreticulin was fused to the 5′ end of DsRed2 and the ER retention sequence, KDEL, was fused to the 3′ end of DsRed2. Plasmid DsRed-Golgi vector was obtained from Clontech (USA), which contains a fusion protein consisting of DsRed-Monomer, a monomeric mutant derived from the tetrameric Discosomasp, red fluorescent protein DsRed, and a sequence encoding the N terminal 81 amino acids of human β 1,4-galactosyltransferase. Plasmids BFP-H-Ras and RFP-H-Ras were constructed by fusing BFP and RFP protein at the N terminus of H-Ras, respectively, and were utilized to mark the plasma membrane. Plasmid containing *orail1* gene, which encodes an essential pore subunit of the CRAC channel on the plasma membrane, was kindly provided by Professor Ding JP at Key Laboratory of Molecular Biophysics, Huazhong University of Science and Technology. For plasmid construction, plasmid Myc-UBIAD1 was tagged with the c-Myc epitope (EQKLISEEDL) at the N terminus of UBIAD1. Plasmid UBIAD1-102 Myc was constructed by inserting the c-Myc epitope at the amino acid 102 of the UBIAD1 protein. For plasmid UBIAD1-Myc, c-Myc epitope was fused at the C terminus of UBIAD1. The full-length UBIAD1 cDNA was subcloned into plasmid EGFP-N1 to make the pUBIAD1-EGFP construct. Plasmids UBIAD1(1-2)-EGFP, UBIAD1(2-2)-EGFP, UBIAD1(3-2)-EGFP, UBIAD1(4-2)-EGFP, UBIAD1(2-1)-EGFP, UBIAD1(3-1)-EGFP, UBIAD1(4-1)-EGFP, UBIAD1(1-1a)-EGFP and UBIAD1(1-1b)-EGFP were constructed by fusing part of UBIAD1 at the 5′ end of EGFP. A series of UBIAD1 deletion clones were made by deleting 20, 40, 45, 50, 55, 60 and 65 amino acids from the N terminus of UBIAD1 and then subcloned into the 5′ end of plasmid EGFP-N1. Orail1-EGFP was constructed by fusing the EGFP at the C terminus of Orail1. UBIAD1-N-Orail1-EGFP was constructed by fusing the UBIAD1 N terminus (the first 80 amino acids) to the N terminus of Orail1-EGFP.

### Site-directed Mutagenesis

PCR-based site-directed mutagenesis was employed to construct a series of UBIAD1-EGFP subclones with specific amino acid mutations. The RPWS motif was sequentially mutated to AAAA, AAWS, APWS, RAWS, RPAA and KPWS, respectively.

### Cell Culture

Human embryonic kidney cell line HEK293, human bladder cancer cell line T24, human prostate cancer cell line PC-3 and human heptocyte cell line L02 were purchased from the American Type Culture Collection (Manassas, VA, USA). HEK293 cells, and L02 cells were cultured with 90% DMEM (Dulbecco’s modified Eagle’s medium) and 10% FBS (fetal bovine serum) in CO_2_ incubator at 37°C (5% CO_2_). PC-3 cells were cultured with 90% F-12 medium and 10%FBS in CO_2_ incubator at 37°C (5% CO_2_). T24 cells were cultured with 90% MEM (Minimum Essential Medium with Eagle’s salts) and 10% FBS in CO_2_ incubator at 37°C (5% CO_2_).

### Cell Transfection

Cells were cultured overnight in 24-well plates without antibiotics in an incubator at 37°C, 5% carbon dioxide (CO_2_). Cell confluency reached 80-90% before nucleofection. 0.8 µg or 0.6 µg plasmid DNA was used for transient cell transfection. The entire cell transfection procedure was performed according to the Lipofectamine^TM^2000 manual.

### Immunohistochemistry (IHC)

HEK293 cells were mounted onto the polylysine-slides and treated with formaldehyde. The slides were blocked and incubated with affinity purified primary antibody (anti-UBIAD1 primary antibody (Abcam) or monoclonal anti-c-Myc (human) antibody (1∶200) (Zymed Laboratories Inc)) overnight at 4°C. For permeabilization of the cells, fixed cells were treated with 0.5% Triton X-100 in PBS (Amresco Inc.) for 20 minutes. Following several washes with PBS, the slides were incubated with FITC-conjugated (green) or R-PE-conjugated (red) secondary antibody (Goat anti-rabbit IgG (H+L) (1∶150) (Protein Tech Group, Inc)) at 37°C for 60 minutes. The fluorescent image was observed under confocal microscopy.

### Cycloheximide and Brefeldin A Treatment

HEK293 cells were transfected with plasmid UBIAD1-EGFP. Following transfection for 24 hours, transfected HEK293 cells were treated with 100 µg/mL cycloheximide for 5 hrs, 10 hrs, and 20 hrs, respectively. For brefeldin A treatment, HEK293 cells transfected with plasmid UBIAD1-EGFP were treated with 5 µg/mL brefeldin A for 0.5 hr, 2 hrs and 5 hrs, respectively.

### Flow Cytometry Assay

Bladder carcinoma T24 cells were treated with wild type and mutant (RPWS→AAAA) UBIAD1, followed by staining with the ANNEXIN V-FITC/PI Apoptosis Assay Kit. Cell count was performed with a Flow Cytometer. Each experiment was repeated three times.

### Extraction of the Golgi Apparatus

0.9×10^9^∼3.5×10^9^ L02 cells were collected, dissolved in homogenate media (0.25 mol/L sucrose, 10 mmol/L Tris-HCL, pH 7.4 with protease inhibitors) and the complexes were homogenized by Dounce. Following a brief centrifugation at 2,000 g for 10 minutes, the post nuclear supernatant (PNS) was collected and laid on the top of the sucrose solution (1.2 mol/L, pH 6.4, including 0.05 mol/L Tris-HCl). Following the sucrose density gradient ultracentrifugation (10,000 g, 4°C) for one hour, the Golgi apparatus was collected by extracting the milky substance between 1.2 mol/L sucrose and PNS. The milky substance was spun down and collected by centrifugation at 12,000 rpm, 4°C for 30 minutes [22].

### Neutral Red Supravital Staining of the Golgi

The above collected Golgi fraction was stained with neutral red dye for 8 to 10 minutes, dropped onto a glass slide and observed under microscope.

### Scanning Electron Microscopy

The extracted Golgi apparatus were further confirmed with observance under a scanning electron microscope (SEM, Tecnai; voltage, 200 kV; Amplification, 25,000x).

### Fluorescent Image Analysis and Statistics

Cells were washed in a chamber with PBS twice and then immersed in PBS for fluorescent imaging. We used a confocal system (FV500, Olympus) and an inverted fluorescent microscopy set (IX81, Olympus) to detect fluorescent signals. C-Myc-UBIAD1, UBIAD1-102-Myc, UBIAD1-Myc and UBIAD1-EGFP were excited by a 488 nm argon ion laser. ER-RFP and Golgi-RFP were excited by a 543 nm He-Ne laser. BFP were excited by a 405 nm diode laser. FLUOVIEW (Olympus) was used as the image acquisition software. Images were acquired, processed, and analyzed with TILL Vision (TILL.Photonics, Germany), Adobe Photoshop (Adobe Systems) and IMAGE J (National Institutes of Health, Public Domain). All experiments were done with three different batches. Every chamber in each batch was normalized.

### Bioinformatics Analyses and Protein Models

A 2-D model of the UBIAD1 transmembrane helices was analyzed and depicted using the bioinformatics program SOSUI [http://bp.nuap.nagoya-u.ac.jp/sosui/, Tokyo University of Agriculture and Technology]. UBIAD1 topology was predicted by the program CPH models 3.0. The Golgi retention signal of UBIAD1 was predicted by the Signal-IP program. The 3-D model of UBIAD1 N terminus was predicted by using Rosetta 3.1 (http://www.rosettacommons.org/manuals/archive/rosetta3.1_user_guide/). Multiple alignments of the protein amino acid sequences was performed by using the Geneious Software (http://www.geneious.com/).

### Statistical Analyses

Data were analyzed using Graphpad Prism software. Results were expressed as the Mean±SD(standard deviation), and the difference was determined by the t test. P value<0.05 was considered significant.

### Analysis of the Golgi Localization Percentage of the Wild Type and Mutant UBIAD1 Proteins

HEK293 cells were transfected with the wild type and mutant UBIAD1-EGFP proteins (Δ50, the first 50 amino acids deleted; Δ55, the first 55 amino acids deleted; AAAA, RPWS→AAAA, RPWS mutated to AAAA; APWS, RPWS→APWS and RPAA, RPWS→RPAA). The number of cells with successful transfection and subsequent expression of the proteins were counted as the total number. Among them, the number of cells with Golgi localization of UBIAD1 was counted. The valid Golgi localization is based upon judging the overlap between the green (the wild type and mutant UBIAD1-EGFP) and the red (the Golgi-RFP marker) images by using the IMAGE J software which can provide the Manders coefficients for the overlap of the images. The degree of the image overlap is determined by the Manders coefficients (with 1 representing complete overlap and 0 representing no overlap) according to user manual of the IMAGE J software. Each protein was counted with three different batches. The mean value±SD(standard deviation) were calculated. The images of the first batch of cells for each protein were provided in the supplementary power point files Figure S1.ppt, Figure S2.ppt, Figure S3.ppt, Figure S4.ppt, Figure S5.ppt and Figure S6.ppt to validate our results. As an example, Manders coefficients for the framed cell in the sample images above (please see each power point file) were derived by using the IMAGE J software and listed in table 1. The detailed numbers were shown in the table 1.

**Table 1:** Golgi localization percentage of the wild type and mutant UBIAD1 proteins.

	Number of cells with Golgi localization of UBIAD1	Total numberof cells	The percentage of Golgi localization (%)	MeanValue (SD)	Manders Coefficients (M1, M2)
**UBIAD1**	30	32	93.8	91.4 (2.4)	(0.992, 0.957)
	57	64	89.1		
	42	46	91.3		
**Δ50**	28	38	73.7	72.2 (2.6)	(0.988, 0.651)
	48	65	73.8		
	27	39	69.2		
**Δ55**	10	29	34.5	25.9 (7.7)	(0.986, 0.988)
	14	59	23.7		
	12	61	19.6		
**AAAA**	1	32	3.1	5.3 (1.9)	(0.969, 0.952)
	2	32	6.3		
	3	47	6.4		
**APWS**	2	26	7.7	8.9 (1.4)	(0.936, 0.985)
	5	58	8.6		
	5	48	10.4		
**RPAA**	37	49	75.5	78.4 (2.9)	(0.965, 0.711)
	36	46	78.3		
	39	48	81.3		

## Results

### UBIAD1 as a Membrane Protein Localized in the Juxta-nuclear Area of the Cytosol

Recently, several groups have independently reported on the subcellular localization of UBIAD1 [11], [12], [13], [15]. Using immunofluorescent microscopy, Nickerson et al. showed that UBIAD1 is localized on mitochondria but not on the endoplasmic reticulum (ER) of the human keratocytes [15]. However, using UBIAD1-EGFP fusion protein, Nakagawa et al. showed that UBIAD1 is localized on the ER rather than the Golgi in human osteoblast-like MG-63 cells [11]. In *Drosophila*, Vos et al. showed that some UBIAD1/Heix is expressed on the mitochondria in S2 cells [12]. In human endothelial cells, Mugoni et al. showed that UBIAD1 is localized in the Golgi membranes [13]. These inconsistent results together with the importance of UBIAD1 as a bladder carcinoma suppressant compelled us to further investigate the subcellular localization of UBIAD1 in human bladder carcinoma cell line T24.

**Figure 1:**
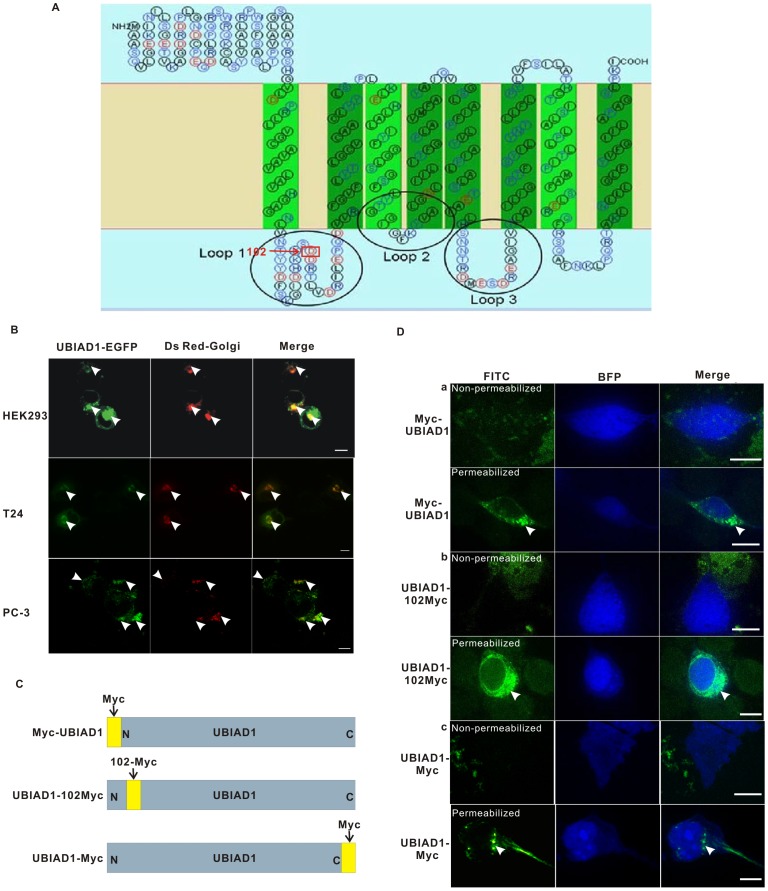
UBIAD1 is accumulated in the juxta-nuclear area inside HEK293, T24 and PC-3 cells. A, Bioinformatics analysis indicates that UBIAD1 is a putative eight transmembrane protein. B, Subcellular localization of UBIAD1 in HEK293, T24 and PC-3 cells are similar. Left, UBIAD1-EGFP; Middle, Ds Red-Golgi (Golgi marker); Right, Merge of left and middle; Upper panel: HEK293 cells; Middle panel: T24 cells; Lower panel: PC-3 cells; Arrows point to UBIAD1-EGFP, the Golgi and the colocalization of UBIAD1 and the Golgi, respectively. C, UBIAD1 was fused with c-Myc-epitope in three different locations (N terminus, amino acid 102 and C terminus). D, UBIAD1 is mainly localized inside the cytosol. a, plasmid Myc-UBIAD1 was transfected into the HEK293 cells; b, plasmid UBIAD1-102 Myc was transfected into the HEK293 cells; c, plasmid UBIAD1-Myc was transfected into the HEK293 cells. pcDNA3.1-BFP was used to mark the whole cell morphology. The HEK293 cells were stained with c-Myc antibody under permeabilized and non-permeabilized conditions, respectively. For permeabilization of the cells, fixed cells were treated with 0.5% Triton X-100 in PBS (Amresco Inc.) for 20 minutes. All experiments were repeated at least three times. Size bar represents 10 µm.

To study its subcellular localization, we first generated a 2-D model of UBIAD1 embedded in lipid membrane using the bioinformatics program SOSUI (Materials and Methods). UBIAD1 is a putative eight transmembrane protein with its N terminus and C terminus located on the same side of lipid membrane (Figure 1A). Based on this model, we generated a construct encoding a fusion protein consisting of UBIAD1 tagged at its carboxyl terminus with an enhanced green fluorescent protein (EGFP). This construct was then used to transfect T24 cells (Figure 1B, middle panel).

Our results showed that, in cultured T24 cell lines, UBIAD1-EGFP was mainly expressed around the juxta-nuclear area inside the cytosol (Figure 1B middle panel). Two types of subcellular localization were observed. One type is a patchy aggregation showing strong fluorescence. The other is a weak fluorescent background throughout the cytosol. Due to the limitation of T24 cells such as low transfection efficiency and the fact that UBIAD1 induces the apoptosis of T24 cells, T24 cells are unsuitable to study subcellular localization and intracellular trafficking of UBIAD1. Human embryonic kidney cell line HEK293 was thus employed.

HEK293 cells were transfected with UBIAD1-EGFP (Figure 1B, upper panel). Subcellular localization similar to that of T24 cells was observed in HEK293 cells for the UBIAD1 protein (Figure 1B, upper panel). The fluorescent images of UBIAD1-EGFP in both T24 and HEK293 cells are similar. A plasmid expressing the Golgi marker Ds Red-Golgi was transfected into both HEK293 and T24 cells (Figure 1B). In both cases, UBIAD1 proteins were observed to colocalize with the Ds Red-Golgi marker, indicating that at least a portion of the UBIAD1 proteins is localized on the Golgi in human bladder carcinoma cells (Figure 1B).

Since UBIAD1 has been shown to affect the growth of human prostate cancer cell line PC-3 [3], PC-3 cells were used to study the subcellular localization of UBIAD1 as well (Figure 1B, lower panel). Plasmid containing UBIAD1-EGFP was used to transfect the PC-3 cells. It is observed that, in PC-3 cells, UBIAD1 is localized in the area similar to that in the T24 and HEK293 cells (Figure 1B, lower panel). UBIAD1 colocalizes with the Golgi in human prostate cancer cell line PC-3.

We further analyzed the subcellular localization of the UBIAD1 N terminus and C terminus using the antiserum against C-Myc epitope. When tagged with the c-Myc epitope (EQKLISEEDL) at the N terminus of UBIAD1, Myc-UBIAD1 was not detected in HEK293 cells under non-permeabilized conditions (Figure 1D, a, upper panel). However, under permeabilized conditions patchy aggregations of UBIAD1 were observed inside the cytosol of the transfected HEK293 cells, indicating the UBIAD1 N terminus was found inside the cytosol. Similar results were obtained when UBIAD1 was tagged with a c-Myc epitope at its C terminus (Figure 1D c). Under non-permeabilized conditions, UBIAD1 was not detected. However, under permeabilized conditions, UBIAD1 was detected inside the cytosol. Again patchy aggregation of UBIAD1 was observed, indicating the UBIAD1 C terminus was found inside the cytosol. The fact that both N terminus and C terminus of UBIAD1 were inside the cytosol is consistent with previously mentioned UBIAD1-EGFP results (Figure 1B).

We then inserted a c-Myc epitope at amino acid 102 of UBIAD1 (Figure 1D b). HEK293 cells were transfected with plasmid UBIAD1-102 Myc. Consistent with the above observations, under non-permeabilized conditions, UBIAD1 was not detected using Myc antibody. In the presence of Triton X-100, cytoplasm localization of UBIAD1 was observed under permeabilized conditions. Again, patchy aggregation of UBIAD1 was observed around the juxta-nuclear area in HEK293 cells, indicating loop 1 at the 102^nd^ amino acid of UBIAD1 is inside the cytosol as well. If UBIAD1 is located on the plasma membrane, and if our proposed topology model is true, then loop 1 would be on the extracellular side and thus would be detectable under non-permeabilized conditions. However, our data showed that loop 1 is detectable only under the permeabilized conditions. Hence, our results suggest that UBIAD1 is a membrane protein inside the cytoplasm, not on the plasma membrane.

### UBIADI is Localized on the Endoplasmic Reticulum (ER) and the Golgi

**Figure 2:**
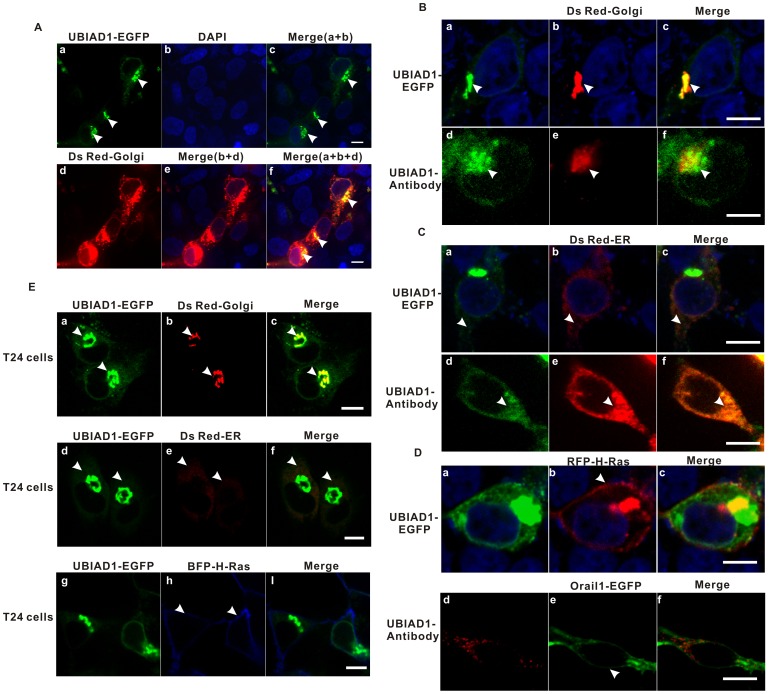
UBIAD1 is localized on the ER (endoplasmic reticulum) and the Golgi, but not on the plasma membrane, of both HEK293 cells and T24 cells. A, UBIAD1 is localized on the Golgi apparatus of HEK293 cells. HEK293 cells were transfected with plasmid UBIAD1-EGFP and plasmid Golgi-RFP (Golgi marker). The nuclei were stained with DAPI. a, UBIAD1-EGFP expressed in HEK293 cells; b, nuclei stained with DAPI; c, the merge of a and b; d, Golgi marker expressed in HEK293 cells; e, the merge of b and d; f, the merge of a, b and d. Arrows point to the UBIAD1-EGFP. B, UBIAD1 is localized on the Golgi of a single HEK293 cell; a, UBIAD1-EGFP expressed in HEK293 cell; Arrows point to the UBIAD1-EGFP. b and e, Ds Red-Golgi marker; Arrows point to the Golgi. c, the merge of a and b; The nuclei in a, b and c were stained with DAPI. d, The endogenous UBIAD1 was measured. HEK293 cell stained with UBIAD1 antibody and the FITC-conjugated secondary antibody; Arrow point to the endogenous UBIAD1. f, the merge of d and e. C, UBIAD1 is localized on the ER of a single HEK293 cell; a, UBIAD1-EGFP expressed in a single HEK293 cell; b and e, Ds Red-ER marker; c, the merge of a and b; The nuclei in a, b and c were stained with DAPI. d, The endogenous UBIAD1 was measured. HEK293 cell stained with UBIAD1 antibody and the FITC-conjugated secondary antibody; f, the merge of d and e. The arrangement of arrows is similar to those in figure 2B. D, UBIAD1 is not detected on the plasma membrane. a, UBIAD1-EGFP expressed in a single HEK293 cell; b, RFP-H-Ras (marker for plasma membrane and the Golgi); Arrow points to plasma membrane. c, the merge of a and b; d, The endogenous UBIAD1 was measured. HEK293 cell stained with UBIAD1 antibody and the R-PE-conjugated secondary antibody; e, Orail1-EGFP (marker for plasma membrane); Arrow points to plasma membrane. f, the merge of d and e. E, As in HEK293 cells, UBIAD1 is expressed on the ER and the Golgi, but not on the plasma membrane, in bladder carcinoma T24 cells. a, d and g, UBIAD1-EGFP expressed in T24 cells; Arrows point to UBIAD1-EGFP. b, e and h, markers for the Golgi (b, Ds Red-Golgi), ER (e, Ds Red-ER) and plasma membrane (h, BFP-H-Ras), respectively; Arrows point to the Golgi, the ER and the plasma membrane. c, Merge of a and b; f, Merge of d and e; i, Merge of g and h. All experiments were repeated at least three times. Size bar represents 10 µm.

The previously mentioned juxta-nuclear patchy aggregation of UBIAD1 suggests that UBIAD1 might be localized on the Golgi. To explore this possibility, HEK293 cells were cotransfected with plasmid UBIAD1-EGFP and Ds Red-Golgi (Golgi marker) followed by staining with DAPI (Figure 2A and 2B a-c). UBIAD1 colocalized with the Golgi marker outside the nucleus, indicating UBIAD1 is localized on the Golgi.

To eliminate the possibility that the Golgi localization of UBIAD1 in HEK293 cells was an artifact of protein over expression, subcellular localization of the endogenous UBIAD1 was studied using the UBIAD1 antibody (Figure 2B d–f). It is clear that endogenous UBIAD1 colocalized with the Golgi marker (Figure 2B–f). Endogenous UBIAD1 is therefore localized on the Golgi as well. In addition, plasmid UBIAD1-EGFP was cotransfected into HEK293 cells with the ER markers (Ds Red-ER) (Figure 2C a–c) and plasma membrane markers (RFP-H-Ras) (Figure 2D a–c), respectively. UBIAD1 also colocalizes with ER (Figure 2C a–c), but not with plasma membrane (Figure 2D a–c). Using Ds Red-ER as the ER marker and Orail1-EGFP as the plasma membrane marker (Materials and Methods), endogenous UBIAD1 can be detected by UBIAD1 antibody on the ER (Figure 2C, d–f), but can not be detected on the plasma membrane (Figure 2D d–f). This matches the UBIAD1-EGFP expression pattern (Figure 2B a–c, 2C a–c and 2D a–c) and further supports the Golgi and ER localization of UBIAD1.

In order to confirm that the above subcellular localization results of UBIAD1 in HEK293 cells also apply to bladder carcinoma cells, we further studied the subcellular localization of UBIAD1 in bladder carcinoma cell line T24. T24 cells were transfected with both UBIAD1-EGFP and subcellular organelle markers (such as Ds Red-Golgi for the Golgi in Figure 2E-b; Ds Red-ER for the ER in Figure 2E-e and BFP-H-Ras for the plasma membrane in Figure 2E-h). As in HEK293 cells, UBIAD1 was present on the Golgi (Figure 2E a–c) and the ER (Figure 2E d–f), but not on the plasma membrane (Figure 2E g–i), in T24 cells. Again, our results showed that the subcellular localization of UBIAD1 in HEK293 and T24 cells are similar.

### UBIAD1 is Localized on the Golgi Apparatus in L02 Cells

**Figure 3:**
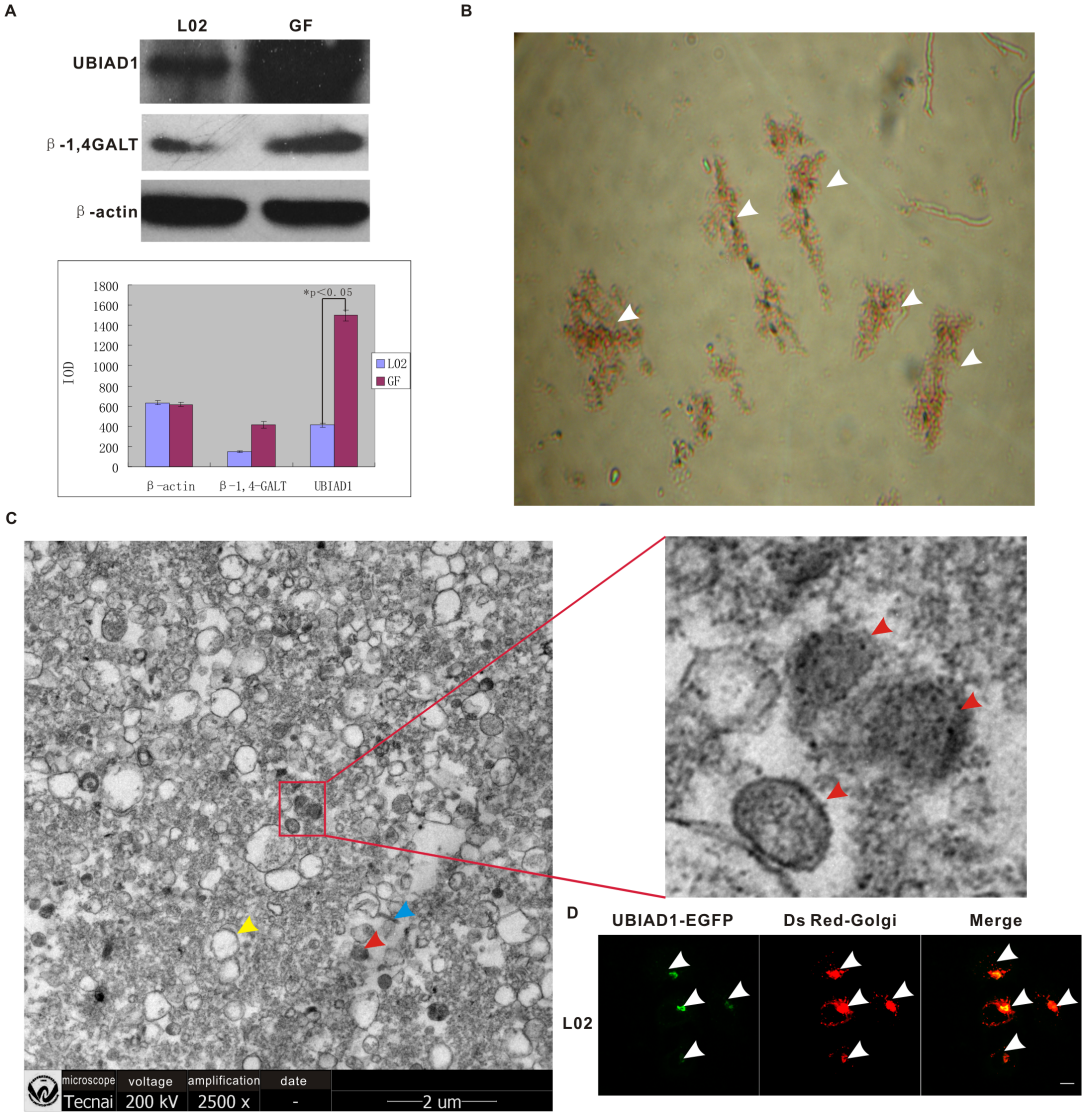
UBIAD1 is localized in the Golgi of L02 cells. A, Proteins were extracted from L02 cells and the isolated Golgi apparatus. The relative intensities of UBIAD1, β1, 4-GALT (Golgi marker) and β-actin bands are shown in the bar graph. IOD (Information Object Definition) was used to specify grayscale images. L02, L02 cells; GF, Golgi fraction; Graph error bars represent standard deviation. Experiments were repeated three times. P<0.05 B, The Golgi apparatus were isolated from the L02 cells and stained with the Golgi-specific neutral red dye by supravital staining. Arrows point to the Golgi fraction. C, Image of the purified Golgi fraction was acquired using Scanning Electronic Microscopy (SEM, 25,000X). Blue arrow, tubular profile; red arrow, cisternae; yellow arrow, secretory vesicle The image of a typical cisternae sectioned tangentially was enlarged on the right [23]. D, UBIAD1-EGFP is accumulated in the Golgi followed by transfection of L02 cells with pUBIAD1-EGFP. Left, UBIAD1-EGFP; Middle, Ds Red-Golgi; Right, merge of UBIAD1-EGFP and Ds Red-Golgi.

In order to further prove that the detected Golgi localization of UBIAD1 is legitimate, Golgi apparatuses were extracted by differential sucrose density gradient ultracentrifugation. Observing that endogenous UBIAD1 is abundantly present in L02 cells, but not in HEK293 cells and bladder carcinoma T24 cells, and noting that the function of UBIAD1 in L02 cells is similar to that in the bladder carcinoma T24 [14], we used L02 cells as the material for isolating the Golgi and extracting large amount of UBIAD1 proteins (Figure 3). The extracted Golgi fractions were then supravitally stained with neutral red dye, a specific dye for the Golgi (Figure 3B). Since the isolated fraction can be stained with neutral red dye, our results verified the isolated fraction as Golgi [23]. To further verify the extracted Golgi fractions as authentic, scanning electron microscopy was used (Figure 3C). Typical Golgi fractions were observed (Figure 3C), composed of cisternae, secretory vesicles and tubular profiles [24]. The image of a typical cisternae sectioned tangentially proves that the extracted material is indeed Golgi apparatuses [24].

Meanwhile, proteins were extracted from the purified Golgi fractions and then separated on SDS-PAGE gel. Western blotting was performed using primary antibodies against UBIAD1, β1, 4-GALT (galactosyltransferase) or β-actin. The detection of the enrichment of β1, 4-GALT, a Golgi specific protein constantly used as the Golgi marker [32], in our purified Golgi fraction once again indicated that the isolated fraction is the Golgi. Our data also showed that UBIAD1 is expressed in L02 cells and is enriched in the extracted Golgi apparatuses (Figure 3A). We also transfected the L02 cells with both pUBIAD1-EGFP and pDsRed-Golgi (Figure 3D). Similar to the situation in T24 cells, UBIAD1 colocalizes with the Golgi marker in L02 cells, suggesting that UBIAD1 is localized on the Golgi apparatuses in human hepatocyte cell line L02, which is consistent with the above results of western blot analysis and scanning electron microscopy. Therefore, endogenous UBIAD1 protein accumulates on the Golgi.

### Subcellular Localization of UBIAD1 on the Golgi is Determined by its N Terminus

**Figure 4:**
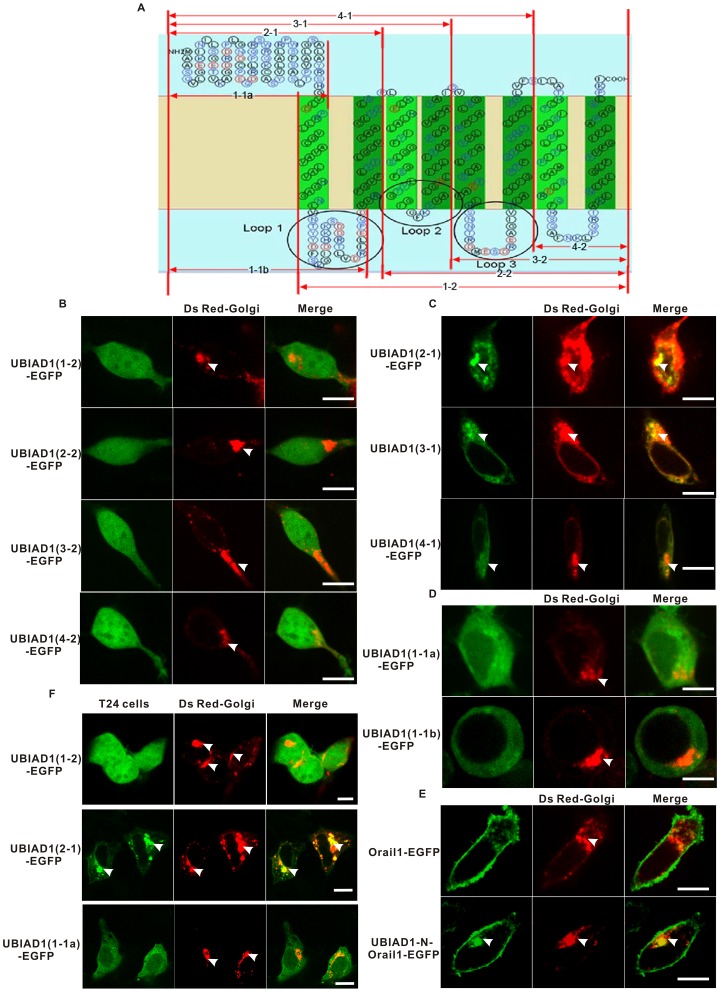
The Golgi retention signal of UBIAD1 resides within its N terminus. A, Schematic representation of the secondary structure of UBIAD1 and the subclones of UBIAD1-EGFP; For example, 1-1a only contains the first 80 amino acids of UBIAD1. B, HEK293 cells were transfected with UBIAD1-EGFP subclones with N terminus deleted (1-2, 2-2, 3-2 and 4-2). C, HEK293 cells were transfected with UBIAD1-EGFP subclones with N terminus remaining (2-1, 3-1 and 4-1) and some of the transmembrane segments deleted. D, HEK293 cells were transfected with UBIAD1-EGFP subclones 1-1a and 1-1b, respectively. E, HEK293 cells were transfected with Orail1-EGFP and UBIAD1-N-Orail1-EGFP respectively. F, Bladder carcinoma T24 cells were transfected with UBIAD1-EGFP subclones 1-2, 2-1, and 1-1a, respectively. The subcellular localizations of these mutant proteins are similar in both HEK293 and T24 cells. All experiments were repeated at least three times. Size bar represents 10 µm.

What is the molecular signal for the retention of UBIAD1 on the Golgi apparatus? To answer this question, a series of subclones were constructed by truncating the full length of UBIAD1-EGFP (Figure 4A). For example, plasmid UBIAD1 (1-2)-EGFP was constructed by deleting the N terminus (the first 80 amino acids) of UBIAD1 and plasmid UBIAD1 (2-1)-EGFP was constructed by keeping the N terminus and the first two transmembrane segments. These subclones were transfected into HEK293 cells and their subcellular localizations were examined with fluorescent microscopy.

Deletion of the UBIAD1 N terminus (the first 80 amino acids) resulted in diffusion of UBIAD1 across the entire HEK293 cell (Figure 4B). This is the reason why 1-2, 2-2, 3-2 and 4-2 mutant protein of UBIAD1-EGFP diffuse across the entire cell since they all lack the UBIAD1 N terminus composed of the first 80 amino acids. Therefore, the retention signal of UBIAD1 on the ER and the Golgi is within its N terminus. However, UBIAD1 (2-1)-EGFP (Figure 4C), which contains the N terminus and the first two transmembrane domains, does localize on the ER and the Golgi. This verifies our finding that the Golgi retention signal of UBIAD1 is located within its N terminus and that the UBIAD1 N terminus plus the first two transmembrane segments is sufficient to retain UBIAD1 on the Golgi. 3-1 and 4-1 mutant proteins of UBIAD1-EGFP can localize on the Golgi since they all contain the UBIAD1 N terminus and the transmembrane segments (Figure 4A and 4C).

We studied the role of transmembrane domain (TMD) in the localization of UBIAD1 on the Golgi. Two subclones containing the N terminus, one (plasmid UBIAD1 (1-1b)-EGFP) with and the other (plasmid UBIAD1 (1-1a)-EGFP) without the first transmembrane domain, were constructed and transfected into HEK293 cells (Figure 4D). It is clear that the transmembrane domain is required for UBIAD1 to localize on the ER and the Golgi. Without it, the N terminus of UBIAD1 would be unable to localize on the ER and the Golgi alone (plasmid UBIAD1 (1-1a)-EGFP). Addition of one transmembrane domain partially restores the ER and Golgi localization of UBIAD1 (Figure 4D, lower panel), suggesting that the transmembrane domain is also necessary for UBIAD1 to localize on the ER and the Golgi, possibly by anchoring UBIAD1 on the lipid membrane.

We also tested whether the N terminus of UBIAD1 can make a non-Golgi membrane protein targeted to the Golgi. For this purpose we used Orail1, an essential pore subunit of the ion channel CRAC [25]. Orail1 is specifically localized on the plasma membrane, as shown by the fluorescent image of Orail1-EGFP (Figure 4E, upper panel). When the N terminus of UBIAD1 was fused with Orail1-EGFP, Orail1 can be targeted to the Golgi (Figure 4E, lower panel). This result indicates that the N terminus of UBIAD1 could provide the information necessary for UBIAD1 to be localized on the Golgi.

The reason why UBIAD1-N-Orail1-EGFP, but not UBIAD1-(1-2)-EGFP, can localize on the Golgi is simply due to the fact that UBIAD1-N-Orail1-EGFP possesses the transmembrane segments required for it to be transported onto the endomembrane system. Due to the lack of transmembrane segments, UBIAD1-(1-2)-EGFP can not be transported onto the endomembrane system and then to the Golgi. Therefore, transmembrane segments are also necessary for localization of UBIAD1 on the Golgi.

Key UBIAD1-EGFP mutant protein such as UBIAD1-(1-2)-EGFP, UBIAD1-(2-1)-EGFP and UBIAD1-(1-1a)-EGFP were also transfected into bladder carcinoma T24 cells (Figure 4F). It is observed that the subcellular localization of these mutant UBIAD1 proteins in T24 cells is similar to those in the HEK293 cells (see above results), indicating that the N terminus of UBIAD1 plays the similar role in the Golgi localization of UBIAD1 in both T24 and HEK293 cells. The results of HEK293 cells are also applicable to the T24 cells.

In conclusion, the Golgi retention signal of UBIAD1 resides within its N terminus. The transmembrane domain might provide an anchor for UBIAD1 to localize on the Golgi lipid membrane.

### The Golgi Retention Signal of UBIAD1

As UBIAD1 localizes on the Golgi, there must be a molecular signal for UBIAD1 to be targeted to and retained on the Golgi apparatus. Based on previous experiments, the Golgi retention signal of UBIAD1 resides within its N terminus.

**Figure 5:**
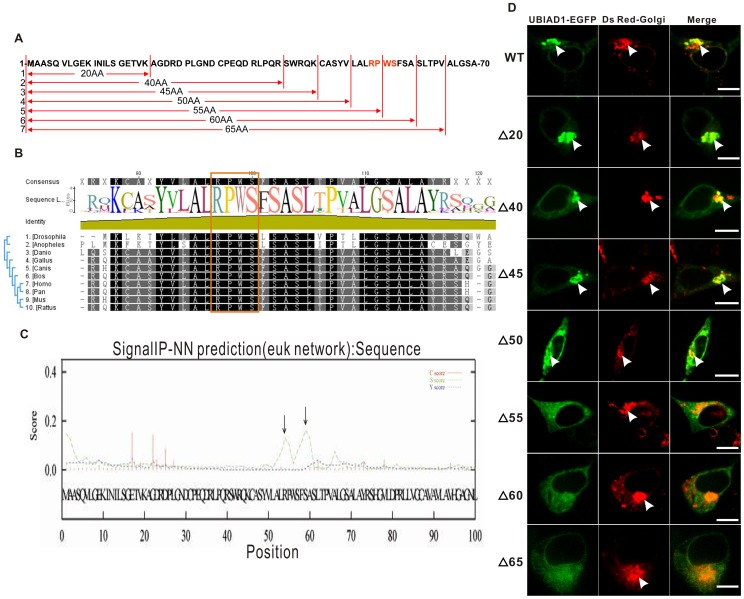
RPWS as the Golgi retention signal of UBIAD1. A, A series of deletion clones were constructed for UBIAD1-EGFP (i.e., 20AA, Δ20, the first 20 amino acids of UBIAD1 N terminus was deleted); B, Multiple alignment of the N terminus of the UBIAD1 putative orthologs from ten different species (including *Drosophila melanogaster, Anopheles gambiae str. PEST, Danio rerio, Gallus gallus, Canis lupus familiaris, Bos Taurus, Homo sapiens, Pan troglodytes, Mus musculus, Rattus norvegicus*) using program Geneious. RPWS is conserved across all ten species; C, RPWS (amino acids 54 to 57) of the UBIAD1 N terminus was predicted to be the Golgi retention signal by Signal IP program. Arrows indicate the predicted Golgi retention signal. D, Deletion clones made in A were sequentially transfected into the HEK293 cells. Arrows point to the Golgi. WT, wild type UBIAD1-EGFP; Δ20, the first 20 amino acids of UBIAD1 N terminus was deleted.

To finely map the exact Golgi retention signal for UBIAD1, a series of deletion clones derived from full-length UBIAD1-EGFP were constructed by sequentially deleting the N terminus (Figure 5A). HEK293 cells were transfected with these deletion clones (Figure 5D). The results show that deletion of the first 45 amino acids has no significant effect on the Golgi localization of UBIAD1. Similar to the wild type UBIAD1 protein (WT), the mutant UBIAD1 protein with 20AA deletion (Δ20), 40AA deletion (Δ40) and 45AA deletion (Δ45) can all localize on the Golgi. The Golgi retention signal, therefore, does not reside within the first 45 amino acids of the N terminus. Deletion of the first 50 or more amino acids (Δ50), however, does affect the localization of UBIAD1 on the Golgi as the amount of UBIAD1 on the ER begins to increase and distinct patchy aggregation of UBIAD1 on the Golgi begins to vanish with longer deleted segments. Deletion of the first 55 amino acids (Δ55) removes the localization of UBIAD1 on the Golgi. UBIAD1 distinctly localizes on the ER, but not on the Golgi. Therefore, at least part of the UBIAD1 Golgi retention signal lies within the amino acids 50–55 of the UBIAD1 N terminus. Deleting the first 55 amino acids disrupts this signal, resulting in the disappearance of UBIAD1 on the Golgi. So do the deletions of the first 60 amino acids (Δ60) and the first 65 amino acids (Δ65).

**Figure 6:**
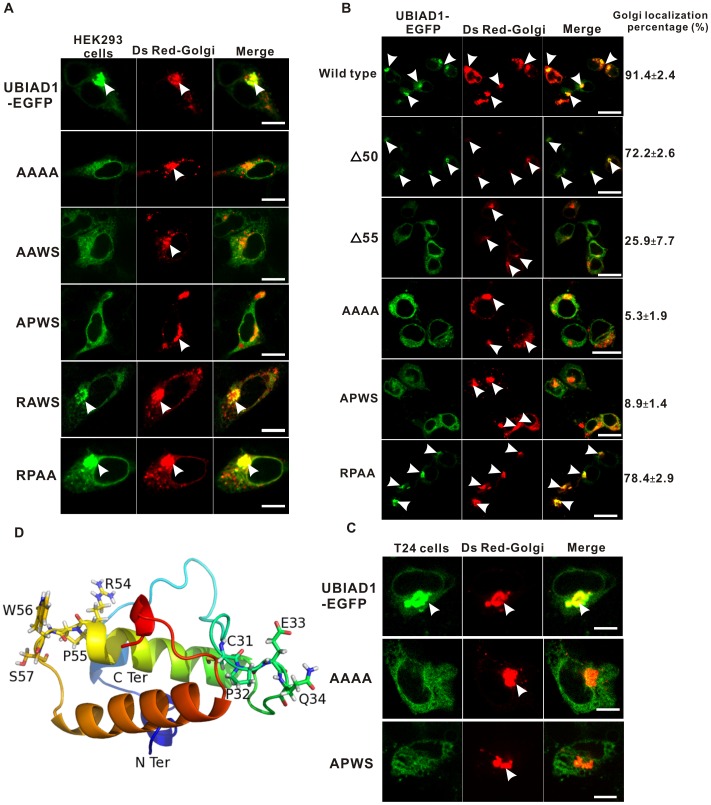
Arginine 54 (R54) as the most crucial amino acid in the UBIAD1 Golgi retention signal RPWS. A, RPWS was sequentially mutated to AAAA, AAWS, APWS, RAWS, and RPAA and was expressed in the HEK293 cells. Arrows point to the Golgi. B, HEK293 cells were transfected with the wild type and mutant UBIAD1-EGFP proteins (Δ50, the first 50 amino acids deleted; Δ55, the first 55 amino acids deleted; AAAA, RPWS→AAAA, RPWS mutated to AAAA; APWS, RPWS→APWS and RPAA, RPWS→RPAA). The percentage of the Golgi localization of each protein is calculated from more than 100 cells (see Table 1 and the supplementary power point files Figure S1.ppt, Figure S2.ppt, Figure S3.ppt, Figure S4.ppt, Figure S5.ppt and Figure S6.ppt for details). Number represents Mean±SD. Experiments were repeated three times. Arrows point to the Golgi and the Golgi localized UBIAD1. C, Bladder carcinoma T24 cells were transfected with full length UBIAD1-EGFP and UBIAD1-EGFP mutant proteins (RPWS→AAAA and RPWS→APWS) D, The predicted 3D structure of the UBIAD1 N terminus by using Rosetta 3.1 (http://www.rosettacommons.org/manuals/archive/rosetta3.1_user_guide/) Size bar represents 10 µm.

We analyzed the N terminus of UBIAD1 with the bioinformatics program Signal IP (Figure 5C). There is a strong Golgi retention signal within amino acids 50–55 of the UBIAD1 N terminus. The result is consistent with the results of our transfected deletion clones. Multiple alignments of UBIAD1 amino acid sequences from ten different species ranging from insect to human showed that the R54-P55-W56-S57 motif is conserved across all ten species (Figure 5B). A tertiary model of the UBIAD1 N terminus was predicted by using the bioinformatics program Rosetta 3.1 (Figure 6). Based on this prediction, the R54-P55-W56-S57 chain served as a unique protein motif which could be the candidate Golgi retention signal.

To determine the responsible amino acid of the RPWS motif for Golgi localization, PCR-based site-directed mutagenesis was used to create mutant clones A54-A55-A56-A57, A54-A55-W56-S57, A54-P55-W56-S57, R54-A55-W56-S57, and R54-P55-A56-A57. HEK293 cells were transfected with these clones and the localization of these mutated proteins were observed (Figure 6A). Mutation of R54 to A54 (clone AAAA, AAWS, APWS) disrupts the Golgi localization of UBIAD1 as UBIAD1 (green) no longer co-localizes with the Golgi (red). Compared to the perfect overlap between the UBIAD1 and the Golgi in wild type UBIAD1, these mutant proteins (R54→A54) can no longer concentrate on the Golgi, but rather spread across the ER, indicating the disruption of the Golgi localization. In contrast, mutation of P55, W56 and S57 to alanine (clones RAWS and RPAA) does not disrupt the Golgi localization of UBIAD1, as mutant proteins can colocalize with the Golgi marker. The amount of mutant proteins (clones RAWS and RPAA) on ER slightly increases, implying that P55, W56 and S57 might contribute to the Golgi localization of UBIAD1, but probably not as crucial as R54.

Statistically, we analyzed the percentage of the Golgi localization for wild type and key UBIAD1 mutant proteins from more than 100 cells for each protein (Figure 6B, please also see Table 1 and the supplementary power point files Figure S1.ppt, Figure S2.ppt, Figure S3.ppt, Figure S4.ppt, Figure S5.ppt and Figure S6.ppt for details). The full length wild type UBIAD1 protein was used as a positive control, showing the highest percentage of the Golgi localization at 91.4%. Deletion of the first 50 amino acids of the UBIAD1 N terminus (Δ50) slightly decreased the percentage of the Golgi localization to 72.2%. Deletion of the first 55 amino acids (Δ55) almost completely abolishes the Golgi localization of UBIAD1. The percentage of the Golgi localization drops to 25.9%, indicating that the UBIAD1 Golgi retention signal lies within the amino acids 50–55 of the UBIAD1 N terminus. For the point mutations, while the RPAA mutant protein still can localize on the Golgi pretty well (78.4%), mutation of R54 to A54 almost completely removes the Golgi localization (with AAAA mutant protein at 5.3% and APWS mutant protein at 8.9%), proving that the Arginine 54 (R54) is the key residue for the Golgi localization of UBIAD1.

To verify that the above results obtained using HEK293 cells are applicable to bladder carcinoma cells, key UBIAD1-EGFP mutant proteins, A54-A55-A56-A57 and A54-P55-W56-S57, were also expressed in the bladder carcinoma T24 cells (Figure 6C). The fluorescent images of these key mutant proteins are the similar to those in the HEK293 cells. Mutation of Arginine 54 (R54) to Alanine disrupts the Golgi localization of the mutant proteins (AAAA and APWS) in T24 cells, indicating that RPWS motif also serves as the Golgi retention signal for UBIAD1 in bladder carcinoma T24 cells.

In summary, the Golgi retention signal of UBIAD1 is the RPWS motif within its N terminus, of which Arginine 54 (R54) is the most crucial amino acid.

### Anterograde Trafficking of UBIAD1 from the ER to the Golgi

**Figure 7:**
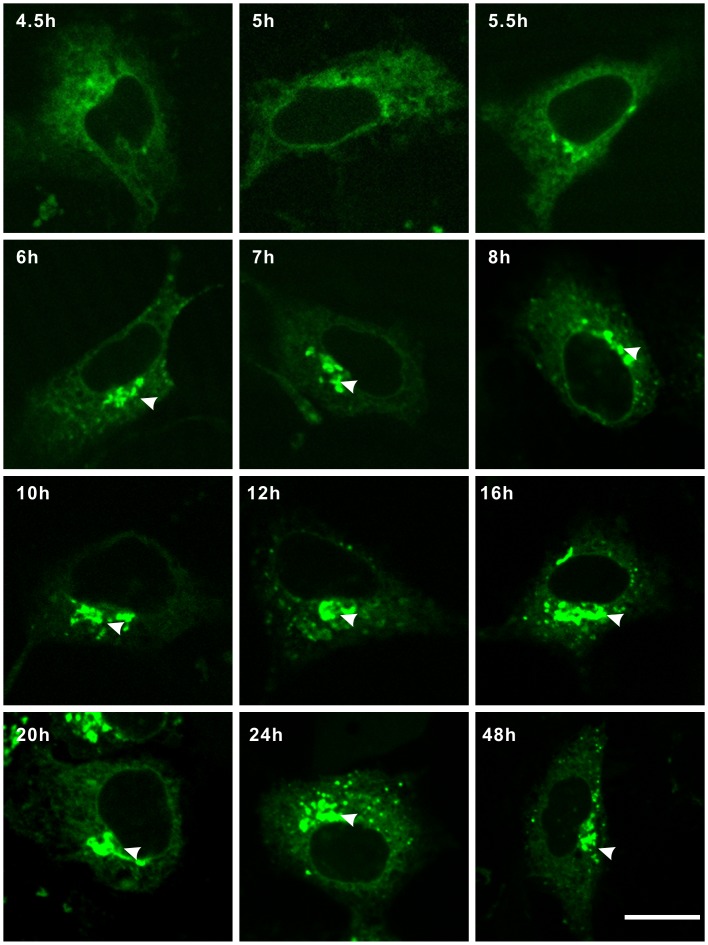
Intracellular trafficking of UBIAD1. UBIAD1 was transported from the ER (endoplasmic reticulum) to the Golgi through anterograde trafficking. HEK293 cells were transfected with UBIAD1-EGFP. Fluorescent images were taken 4.5 hr, 5 hr, 5.5 hr, 6 hr, 7 hr, 8 hr, 10 hr, 12 hr, 16 hr, 20 hr, 24 hr and 48 hr following the transfection. Arrows point to the Golgi. All experiments were repeated three times. Size bar represents 10 µm.

As UBIAD1 resides on the endoplasmic reticulum (ER) and the Golgi in HEK293 cells, it is likely that UBIAD1 is first synthesized on the ER and then targeted to the Golgi via anterograde trafficking. To test this hypothesis, plasmid UBIAD1-EGFP was transfected into HEK293 cells. The subcellular localization of UBIAD1 was observed from 4.5 hours to 48 hours following the transfection (Figure 7). We observed UBIAD1 first appearing on the ER after 4.5 hours of transfection. Between 4.5 and 5 hours after transfection, UBIAD1 was observed to be concentrated on the ER and not on the Golgi. After 5.5 hours following transfection, trace amounts of UBIAD1 began to appear on the Golgi. After 6 hours following transfection, UBIAD1 began to accumulate on the Golgi. After 10 hours following transfection, significant amounts of UBIAD1 proteins accumulated on the Golgi. The amount of UBIAD1 proteins on the ER and on the Golgi began to reach a balance. Between 10 to 48 hours following transfection, the steady-state levels of UBIAD1 on the ER and the Golgi stabilized. It is therefore reasonable to conclude that UBIAD1 is first synthesized on the ER and then targeted to the Golgi. Furthermore, it seems that there is some kind of retention mechanism which keeps the steady-state level of UBIAD1 abundant on the Golgi apparatus.

**Figure 8:**
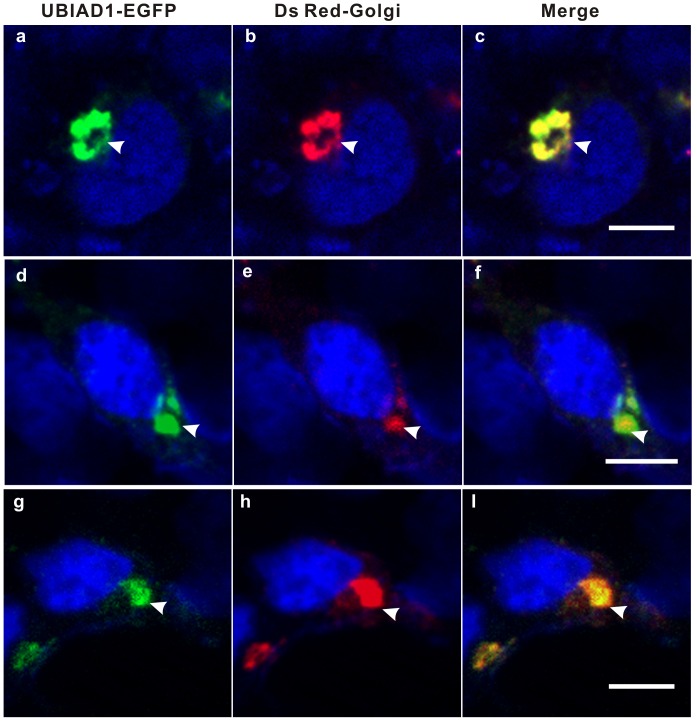
UBIAD1 was strongly retained on the Golgi. HEK293 cells were transfected with UBIAD1-EGFP. Following 20 hours of transfection, the HEK293 cells were treated with cycloheximide (100 ug/mL) for 5 hrs (a–c), 10 hrs (d–f) and 20 hrs (g–i), respectively. Nuclei of the HEK293 cells were stained with DAPI. Arrows point to the Golgi. All experiments were repeated three times. Size bar represents 10 µm.

To further investigate whether UBIAD1 can be driven out of the Golgi, cycloheximide was used to block UBIAD1 synthesis (Figure 8). HEK293 cells were transfected with plasmid UBIAD1-EGFP. Following 20 hours of transfection, the transfected HEK293 cells were treated with cycloheximide for 5 hours (Figure 8, a–c), 10 hours (Figure 8, d–f), and 20 hours (Figure 8, g–i), respectively. It seems that cycloheximide treatment completely drives out the UBIAD1 protein from the ER, even at very short period of time treatment (5 hours, Figure 8 a–c), implying that UBIAD1 was not retained on the ER very well. On the other hand, UBIAD1 adheres to the Golgi apparatus tightly and is hard to be driven out of the Golgi by cycloheximide treatment, even at treatment of long period of time (Figure 8 g–i), further suggesting that UBAID1 is mainly retained on the Golgi.

### Anterograde Trafficking of UBIAD1 from the ER to the Golgi Probably Depends on a COPII-mediated Mechanism

**Figure 9:**
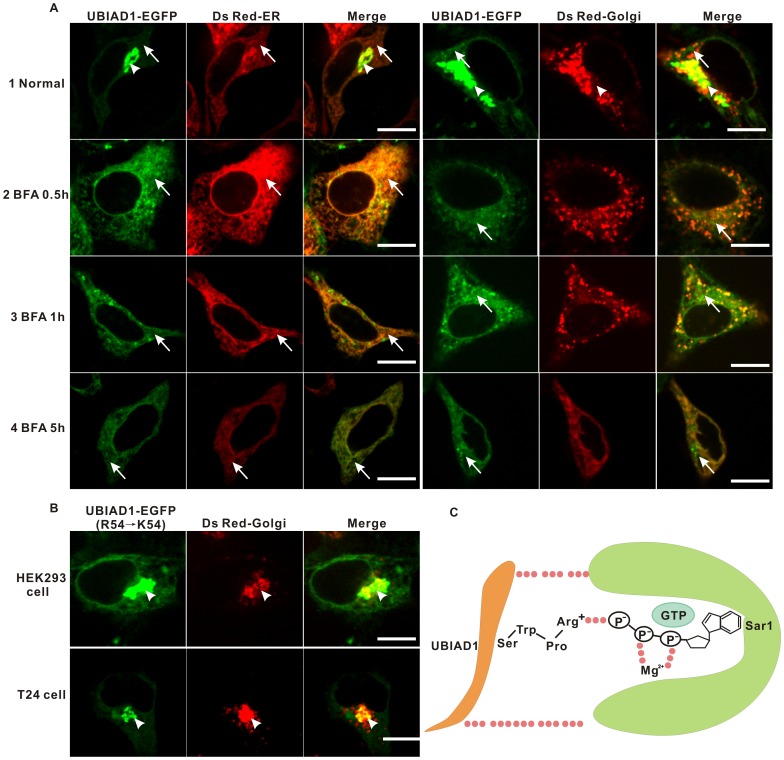
UBIAD1 might be targeted to the Golgi by COPII-mediated mechanism. A, HEK293 cells were transfected with pUBIAD1-EGFP and co-transfected with Ds Red-ER (ER marker) and Ds Red-Golgi (Golgi marker), respectively. Following 24 hours of transfection, transfected HEK293 cells were treated with DMEM (1normal, as control) and brefeldin A (BFA, 5 ug/mL) for 0.5 hr (2), 1 hour (3) and 5 hours (4). Line arrow: ER; Arrow head: the Golgi. B, Substitution of Arginine 54 (R54) to lysine (K) does not affect the Golgi localization of UBIAD1. UBIAD1-EGFP was site-directed mutagenised by substituting the Arginine 54 (R54) to lysine. The mutant clone was transfected into the HEK293 and T24 cells. C, A model representing the putative interactions between RPWS of the UBIAD1 and the GTP of the Sar1 subunit of the COPII complex; Dotted lines represent the possible interactions. Experiments were repeated at least three times. Size bar represents 10 µm.

Eukaryotic secretory proteins are transported from the the endoplasmic reticulum (ER) to the Golgi via coat protein complex II (COPII) proteins [26]. As UBIAD1 exits the ER and then appears on the Golgi, we suspect that a COPII-mediated mechanism is probably utilized to transport UBIAD1 from the ER to the Golgi. To test this hypothesis, HEK293 cells transfected with plasmid UBIAD1-EGFP for 20 hours (Figure 9A1) were treated with a COPII complex inhibitor brefeldin A (BFA) (Figure 9A2-4), which blocks the action of COPII vesicles thereby preventing anterograde transport [27]. Based on our experiments, 0.5 hour treatment of BFA (Figure 9A 2) changes the morphology of HEK293 cells. Following treatment the portion of UBIAD1 proteins localized to the Golgi apparatus starts to decrease while the portion of UBIAD1 on the ER starts to increase, indicating the inhibition of anterograde trafficking of UBIAD1 from the ER to the Golgi. One hour after treating with BFA (Figure 9A 3), the morphology of HEK293 cells begins to change back to normal. However, the accumulation of UBIAD1 on the Golgi does not increase back to its normal level. The amount of UBIAD1 on the ER continues to increase. After 5 hours following treatment with BFA, the morphology of HEK293 cells has almost completely changed back to normal. The localization of UBIAD1 on the Golgi is completely removed (Figure 9A 4). Most UBIAD1 proteins reside on the endoplasmic reticulum (ER). Therefore, disruption of the COPII complex formation impedes the anterograde trafficking of UBIAD1 from the ER to the Golgi. UBIAD1 is transported to the Golgi from the ER via a COPII-mediated mechanism.

The recognition of cargo from the ER by COPII protein complexes depends on the small GTPase protein Sar1 and Sec24 [28], [29]. As the Golgi retention signal of UBIAD1 relies on the RPWS motif, of which Arginine 54 is the most important, it is reasonable to suspect that the UBIAD1 RPWS motif serves as the arginine finger recognized by Sar1 proteins (Figure 9C) [30]. S57-W56-P55-R54 could serve as a decent P-loop that recognizes the GTP of Sar1 proteins [31]. If this is the case, mutation of R54 to lysine (K54), would not affect the recognition of Sar1 proteins by this P-loop as lysine could provide the positively charged amino group necessary to stabilize the negatively charged GTP of Sar1. To test this hypothesis, PCR-based site-directed mutagenesis was used to construct a UBIAD1-EGFP clone with R54 mutated to K54 (KPWS). Both HEK293 and T24 cells were transfected with KPWS mutant and fluorescent images were taken. Compared to the APWS mutant (Figure 6), KPWS mutant protein can localize on the Golgi pretty well (Figure 9B) in both HEK293 and T24 cells. The results show that mutation of R54 to K54 does not affect the localization of UBIAD1 on the Golgi, thus verifying our model convincingly. The results further implies that the intracellular trafficking mechanism of UBIAD1 is similar in HEK293 and T24 cells.

### Golgi Localization of UBIAD1 Influences its Tumor Suppressing Activity

Subcellular localization of protein determines its biological function. As UBIAD1 is a bladder carcinoma suppressor, it is reasonable to presume that the subcellular localization of UBIAD1 on the Golgi is related to its tumor suppressing function.

**Figure 10:**
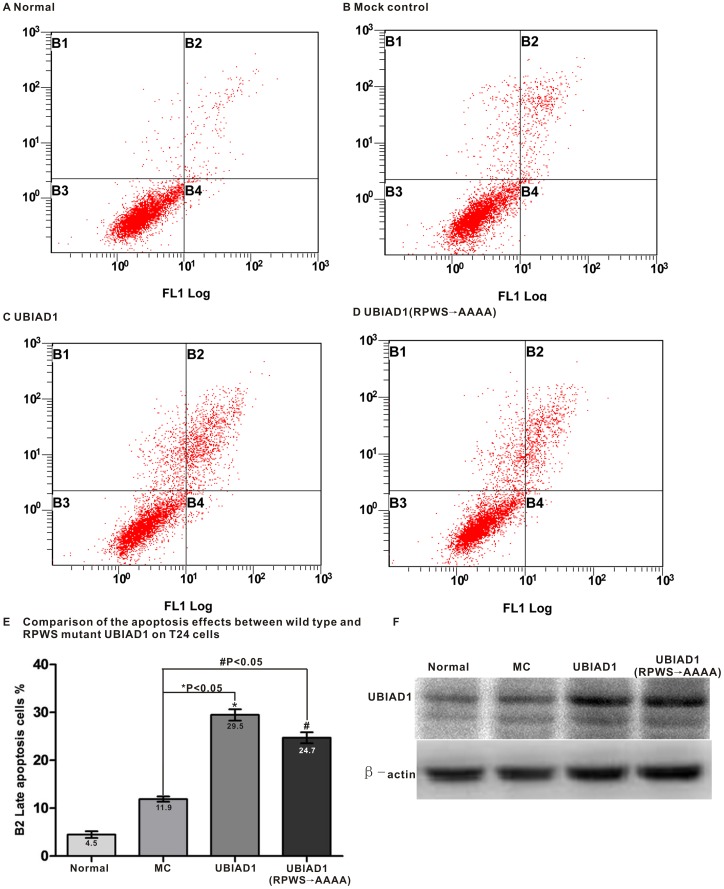
Localization of UBIAD1 on the Golgi influences its tumor suppressing activity. The bladder carcinoma T24 cells were treated with wild type and mutant (RPWS→AAAA) UBIAD1, followed by the staining with the ANNEXIN V-FITC/PI Apoptosis Assay Kit. Cell counts were performed with a flow cytometer. A, Normal, T24 cells stained with FITC and PI; B, Mock control, T24 cells treated with Lipofectamine 2000 (stained with FITC and PI 48 hr later); C, UBIAD1, T24 cells transfected with pcDNA3.1-UBIAD1 (stained with FITC and PI 48 hr later); D, UBIAD1(RPWS→AAAA), T24 cells transfected with (RPWS→AAAA) mutant of UBIAD1 with RPWS changed to AAAA (stained with FITC and PI 48hr later); E, Comparison of the apoptosis effects (B2) between wild type and (RPWS→AAAA) mutant UBIAD1 on T24 cells. Numbers were expressed as Mean±SD, which is represented as graphing error bar. Normal: 4.5±0.7%, MC: 11.9±0.5%, UBIAD1∶29.5±1.2%, UBIAD1 (RPWS→AAAA): 24.7±1.2%. *P = 0.0002<0.05, #P = 0.0006<0.05. Each experiment was repeated three times. B1: Mechanically damaged cells; B2: Late apoptosis cells; B3: Normal cells; B4: Early apoptosis cells; F, Western blot analysis of the amount of UBIAD1 protein in T24 cells. Normal, MC, UBIAD1 and UBIAD1 (RPWS→AAAA) are as above. In all of the above wild type and mutant UBIAD1 proteins, no GFP is attached.

To explore this connection, we tested the effect of removing the Golgi localization of UBIAD1 on its tumor suppressing activity. Bladder carcinoma T24 cells were transfected with wild type UBIAD1 and UBIAD1 RPWS→AAAA mutant (with RPWS changed to AAAA) with no GFP attached. ANNEXIN V-FITC/PI Apoptosis Assay Kit was used to detect cell apoptosis (Figure 10). Normal T24 cells were used as the control group (Figure 10 A, B). It seems that the addition of UBIAD1 induces apoptosis of bladder carcinoma T24 cells with percentages of late apoptosis cells (B2) increasing from 11.9% (MC, mock control, T24 cells with Lipofectamine only) to 29.6% (Figure 10E). Compared to wild type UBIAD1, addition of UBIAD1 RPWS→AAAA mutant protein into T24 cells produced a smaller percentage of late apoptosis cells (24.7% for RPWS→AAAA mutant vs. 29.5% for wild type UBIAD1). The experiment was repeated three times. Each time, UBIAD1 RPWS→AAAA mutant protein caused fewer amounts of late apoptosis cells. To make sure that the apoptosis of T24 cells was induced by the addition of UBIAD1 protein, the amount of UBIAD1 protein in the above four experiments was analyzed by the western blot (Figure 10F). It is clear that the amount of endogenous UBIAD1 protein in T24 cells is scarce. Transfections of pcDNA3.1-UBIAD1 and pcDNA3.1-UBIAD1 (RPWS→AAAA) mutant into the T24 cells increase the amount of wild type and mutant UBIAD1 protein, respectively. The apoptosis of T24 cells is indeed induced by the UBIAD1 protein.

In conclusion, disrupting the Golgi localization of UBIAD1 decreases the percentage of UBIAD1-induced late apoptosis cells, indicating that the tumor suppressing activity of UBIAD1 protein is at least partially influenced by its proper Golgi localization.

## Discussion

UBIAD1 has been shown to be an important disease-related protein in both dyslipidemia associated SCD (Schnyder’s corneal dystrophy) and bladder carcinoma [2], [4]. It has been shown that UBIAD1 is a key enzyme in vitamin K (MK-4) biosynthesis and is also involved in the neurodegenerative Parkinson’s disease [11], [12]. Recently, UBIAD1 has been shown to be a CoQ10 synthase localized in the Golgi membranes associated with cardiovascular disease [13]. So far, reports on the subcellular localization of UBIAD1 have been inconsistent. In this paper, we examined the subcellular localization of UBIAD1 in human bladder carcinoma cell line T24 as well as in human prostate cancer cell line PC-3, human embryonic kidney cell line HEK293 and human hepatocyte cell line L02. We showed that UBIAD1 is a transmembrane protein that accumulates on the Golgi apparatus of human bladder carcinoma cells. A novel Golgi retention signal, RPWS, was identified, which affects the tumor suppressing activity of UBIAD1.

### The subcellular Localization of UBIAD1

Subcellular localization of protein play a vital role in the proper function of many membrane proteins, e.g. the Ras protein [21]. Our results suggest that UBIAD1 is a transmembrane protein distributed on the endoplasmic reticulum (ER) and the Golgi apparatus. The steady-state level of UBIAD1 is most abundant on the Golgi apparatus.

It has been shown using immunofluorescent microscopy that UBIAD1 colocalizes with OXPHOS protein complex I, an enzyme found in mitochondria [15]. However, UBIAD1 was not observed to colocalize with the protein disulfide isomerase, an enzyme located on the endoplasmic reticulum (ER). In *Drosophila* as well, it has been shown that at least part of UBIAD1/Heix localizes on the mitochondria of S2 cells [12]. Localization of UBIAD1 on the mitochondria is surprising considering a previous report that demonstrated the protein-protein interaction between UBIAD1 and apolipoprotein E, which has not been found to localize on the mitochondria [9].

The discrepancy between our results and the results from other groups may be due to the following explanations. Different cell lines could yield different subcellular localization results. In the study of Nickerson et al, human keratocytes were used to study the subcellular localization of UBIAD1 whereas in our study human bladder carcinoma cell line T24 as well as prostate cancer cell line PC-3, embryonic kidney cell line HEK293 and hepatocyte cell line L02. were used instead. The different cell lines studied might have contributed to the different staining patterns. As UBIAD1 might exert different functions on different cell organelles, by locating UBIAD1 in specific intracellular organelles, different cells can achieve specialized functions.

We must also note that the result of UBIAD1 mitochondrial localization does not necessarily contradict our results. In our experiments we also observed juxta-nuclear staining outside the Golgi apparatus (Figure 2B d–f), which could represent other intracellular organelles, including the mitochondria. In *Drosophila* S2 cells, UBIAD1/Heix has been observed to localize on extracellular organelles besides the mitochondria (Figure 4A, 4B in [12]). In addition, Nickerson et al. concluded that they could not exclude the staining of other intracellular organelles [15]. Therefore, UBIAD1 could localize on the ER, the Golgi and the mitochondria. Depending upon different cell types, the steady-state level of UBIAD1 on these intracellular organelles may vary. The fact that the colocalization signal of the endoplasmic reticulum is faint in our experiment (Figure 2C a-c, Figure 2E d–f) may also explain the absence of endoplasmic reticulum staining in the results of the other group [15].

Nakagawa et al. reported the colocalization between UBIAD1 and the endoplasmic reticulum, which is consistent with our results [11]. However, they did not detect the colocalization of UBIAD1 with the Golgi apparatus, which might be due to the osteoblast-like MG-63 cells they used. Interestingly, Mugoni et al. recently showed [13] that UBIAD1 is localized in Golgi membranes in human endothelial cells, which is consistent with our results.

### RPWS, an Arginine Finger, Serves as a New Golgi Localization Signal for UBIAD1

Many different molecular mechanisms may contribute to the protein retention on the Golgi apparatus [32]. For example, the Vps74p/GOLPH3-mediated retention mechanism contributes to the retention of type II proteins on the Golgi [33], [34]. Among these different mechanisms, signal-based retention of Golgi resident proteins via Amino-Terminal Arginine-Based motifs could contribute to the retention of Och1p and Mnn9p, yeast glycosyltransferases, on the Golgi [17], [35], [36]. These arginine-based motifs are usually composed of two or more arginine residues such as -RR-, -RXR- and –RRR etc. [32].

Our results showed that RPWS motif (54–57) of the UBIAD1 N terminus is the Golgi retention signal of UBIAD1 (Figure 6D) and Arginine 54 (R54) of the RPWS motif makes the most crucial contribution to the Golgi localization of UBIAD1. Prediction of the tertiary structure for the UBIAD1 N terminus indicates that the RPWS amino acid stretch forms a unique protein motif on one end of the N terminus (Figure 6D). This tertiary motif is conserved across all eukaryotic species examined (from *Drosophila* to human, Figure 5B). The tertiary structure of this motif resembles the P loop of arginine finger Phe723-Pro53-Arg722-P-loop in the Sec23 protein of COPII protein complexes [37]. As the amino acid structure of Trp (W) is similar to that of Phe (F), the RPWS tertiary structure may form a natural P loop similar to the one found in Sec23. The P loop of UBIAD1 may be recognized by the GTP of Sar1 [30]. There could be many other protein-protein interactions existing between UBIAD1 and Sar I (Figure 9C). However, the interaction between RPWS of the UBIAD1 and GTP of the Sar I is probably employed to recognize the cargo, UBIAD1 protein, by the COPII complex. Moreover, since GFP in SarI is important for the initiation of the COPII complex formation, recognition of the Sar I GTP by the RPWS motif of the UBIAD1 may trigger this initiation where UBIAD1, as cargo, is recognized and picked up by a COPII complex and delivered to the Golgi (Figure 9C).

### Anterograde Trafficking of UBIAD1 from the ER to the Golgi may be Mediated by COPII Protein Complex

Proteins need to be targeted to specific location to perform distinct biological function. Intracellular hydrophobic proteins, especially secretory proteins, need to be transported by the endoplasmic membrane system to different venues for their specific function. As UBIAD1 is distributed on the ER and the Golgi, it is probably synthesized on the ER and transported to the Golgi apparatus through anterograde trafficking.

We observed that UBIAD1 is transported via the endomembrane system from the ER to the Golgi (Figure 7). Disrupting the ER localization of UBIAD1 completely disrupts the Golgi localization of UBIAD1 (data not shown), indicating its synthesis on the ER and subsequent targeting to the Golgi apparatus by anterograde trafficking. Since the treatment of brefeldin A (BFA) blocks the transport of the UBIAD1 protein from the ER to the Golgi (Figure 9), the anterograde trafficking of UBIAD1 from the ER to the Golgi might be mediated by the COPII protein complex.

The COPII vesicle formation is initiated by the GDP-GTP exchange on Sar1 catalyzed by transmembrane guanine nucleotide exchange factor Sec12 [37], [38]. Activated Sar1-GTP binds to the ER membrane and recruits the Sec23/24 subcomplex. The cytoplasmically expressed signal of transmembrane cargo is then captured through direct contact with Sec24, forming the “prebudding complex” [39]. It is currently not clear whether the membrane-bond Sar1-GTP associates with cargo before the recruitment of Sec23/24 or if the lateral diffusion of Sar1-GTP-Sec23/24 completes cargo capture. It was reported that Sar1-GTP bound to immobilized glycosyltransferase binds Sec23P, implying Sar1 GTP first recognizes cargo and then recruits Sec23/24 protein complex [40].

In the case of UBIAD1, the arginine finger RPWS within the UBIAD1 N terminus forms a potent P-loop (S57-W56-P55-R54), which could interact with GTP on the Sar1 subunit to stabilize the interaction between UBIAD1 and small Sar1 GTPase (Figure 9C) [31]. This protein complex may then recruit the Sec23/24 protein complex. The arginine finger of Sec23 could substitute for the arginine finger of UBIAD1 and Sec24 could bind to UBIAD1 though a protein-protein interaction such that UBIAD1 is recruited by the COPII pre-budding complex and delivered from the ER to the Golgi through anterograde trafficking.

In conclusion, UBIAD1 is localized on the ER and the Golgi in human bladder carcinoma cells T24. It is transported from the ER to the Golgi by anterograde trafficking. The UBIAD1 Golgi retention signal is the RPWS motif within which its N terminus and arginine 54 is the most crucial amino acid for the Golgi localization. The anterograde trafficking of UBIAD1 from the ER to the Golgi may be mediated by the COPII protein complex. Disrupting the Golgi localization of UBIAD1 affects its tumor suppressing activity, suggesting that Golgi localization of UBIAD1 influences its biological function as a tumor suppressor. The fact that disruption of the Golgi localization of UBIAD1 only partially affects its tumor suppressing activity implies that the subcellular localization of UBIAD1 on other intracellular organelles such as ER or mitochondria may also contribute to its tumor suppressing activity. The exact molecular mechanism behind this waits for the future exploration. Overall, our study paves the way for further understanding the molecular mechanism of UBIAD1, a tumor suppressor involved in the biosynthesis of vitamin K2 which is capable of inducing the apoptosis of cancer cells [41].

## Supporting Information

Figure S1 **Wild type UBIAD1 localization in HEK293 cells.** Arrows point to the colocalization of UBIAD1-EGFP and the Golgi. Bar represents 10 µm. Manders coefficients (M1 = 0.992, M2 = 0.957, for details please see Materials and Methods) were derived by using the IMAGE J software (using the framed cell as an example).
(PPT)

Figure S2 **Localization of UBIAD1 mutant Δ50AA in HEK293 cells.** Arrows point to the colocalization of UBIAD1-EGFP (Delta 50AA) and the Golgi. Bar represents 10 µm. Manders coefficients (M1 = 0.988, M2 = 0.651) were derived by using the IMAGE J software (using the framed cell as an example).
(PPT)

Figure S3 **Localization of UBIAD1 mutant Δ55AA in HEK293 cells.** Arrows point to the colocalization of UBIAD1-EGFP (Delta 55AA) and the Golgi. Bar represents 10 µm. Manders coefficients (M1 = 0.986, M2 = 0.988) were derived by using the IMAGE J software (using the framed cell as an example).
(PPT)

Figure S4 **Localization of UBIAD1 mutant AAAA in HEK293 cells.** Arrows point to the colocalization of UBIAD1-EGFP (AAAA) and the Golgi. Bar represents 10 µm. Manders coefficients (M1 = 0.969, M2 = 0.952) were derived by using the IMAGE J software (using the framed cell as an example).
(PPT)

Figure S5 **Localization of UBIAD1 mutant APWS in HEK293 cells.** Arrows point to the colocalization of UBIAD1-EGFP (APWS) and the Golgi. Bar represents 10 µm. Manders coefficients (M1 = 0.936, M2 = 0.985) were derived by using the IMAGE J software (using the framed cell as an example).
(PPT)

Figure S6 **Localization of UBIAD1 mutant RPAA in HEK293 cells.** Arrows point to the colocalization of UBIAD1-EGFP (RPAA) and the Golgi. Bar represents 10 µm. Manders Coefficients (M1 = 0.965, M2 = 0.711) were derived by using the IMAGE J software (using the framed cell as an example).
(PPT)
