# Supplementary figures and images for: A Novel Golgi Retention Signal RPWS for Tumor Suppressor UBIAD1
Source: PLoS One. 2013 Aug 19;8(8):e72015. doi: 10.1371/journal.pone.0072015 (PMC3747158; doi:10.1371/journal.pone.0072015)

## Slide 1
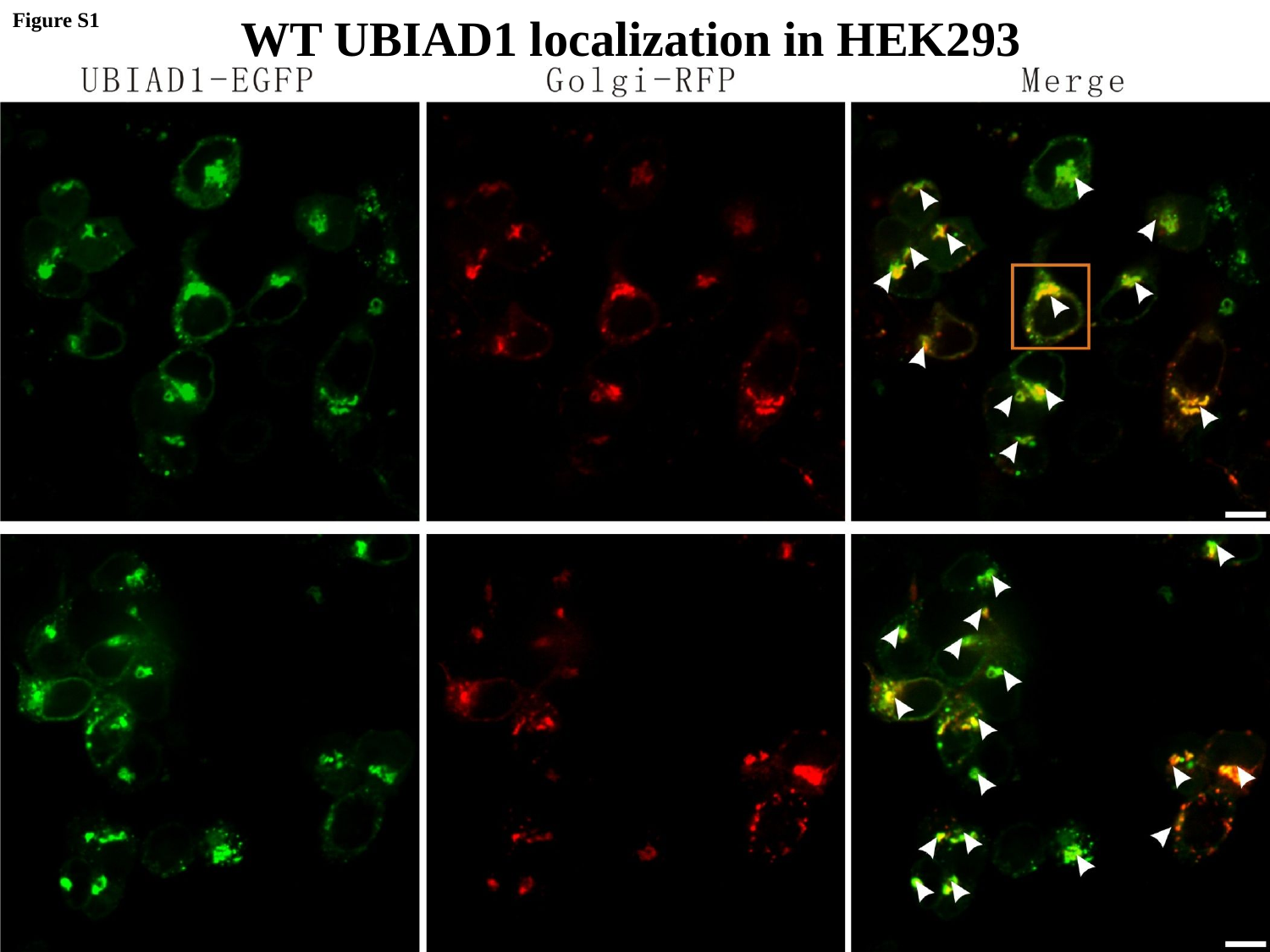

Figure S1
WT UBIAD1 localization in HEK293

Supplement: Figure S1 — Wild type UBIAD1 localization in HEK293 cells. Arrows point to the colocalization of UBIAD1-EGFP and the Golgi. Bar represents 10 µm. Manders coefficients (M1 = 0.992, M2 = 0.957, for details please see Materials and Methods) were derived by using the IMAGE J software (using the framed cell as an example). (PPT) [file pone.0072015.s001.ppt]

## Slide 1
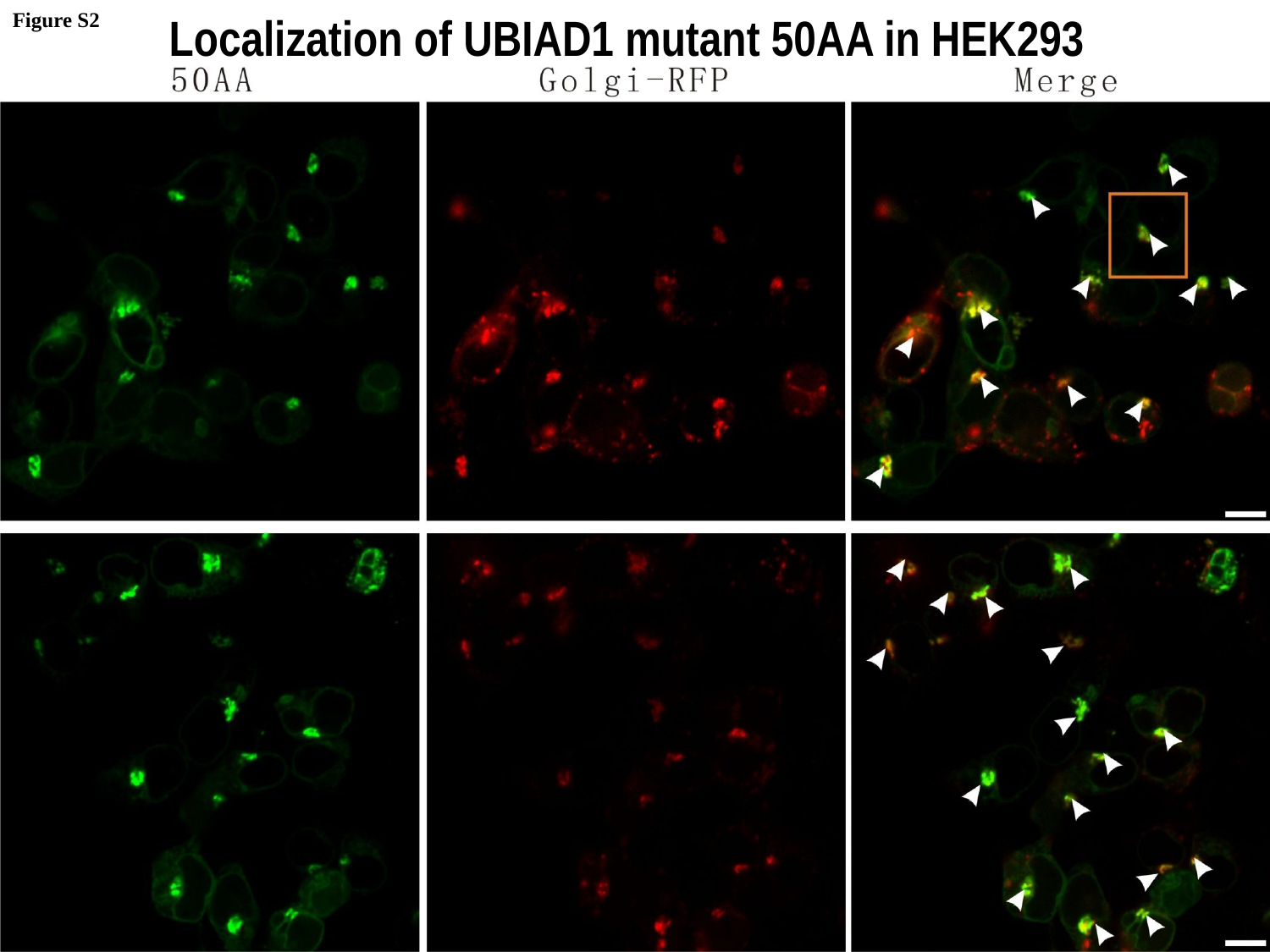

Figure S2
Localization of UBIAD1 mutant 50AA in HEK293

Supplement: Figure S2 — Localization of UBIAD1 mutant Δ50AA in HEK293 cells. Arrows point to the colocalization of UBIAD1-EGFP (Delta 50AA) and the Golgi. Bar represents 10 µm. Manders coefficients (M1 = 0.988, M2 = 0.651) were derived by using the IMAGE J software (using the framed cell as an example). (PPT) [file pone.0072015.s002.ppt]

## Slide 1
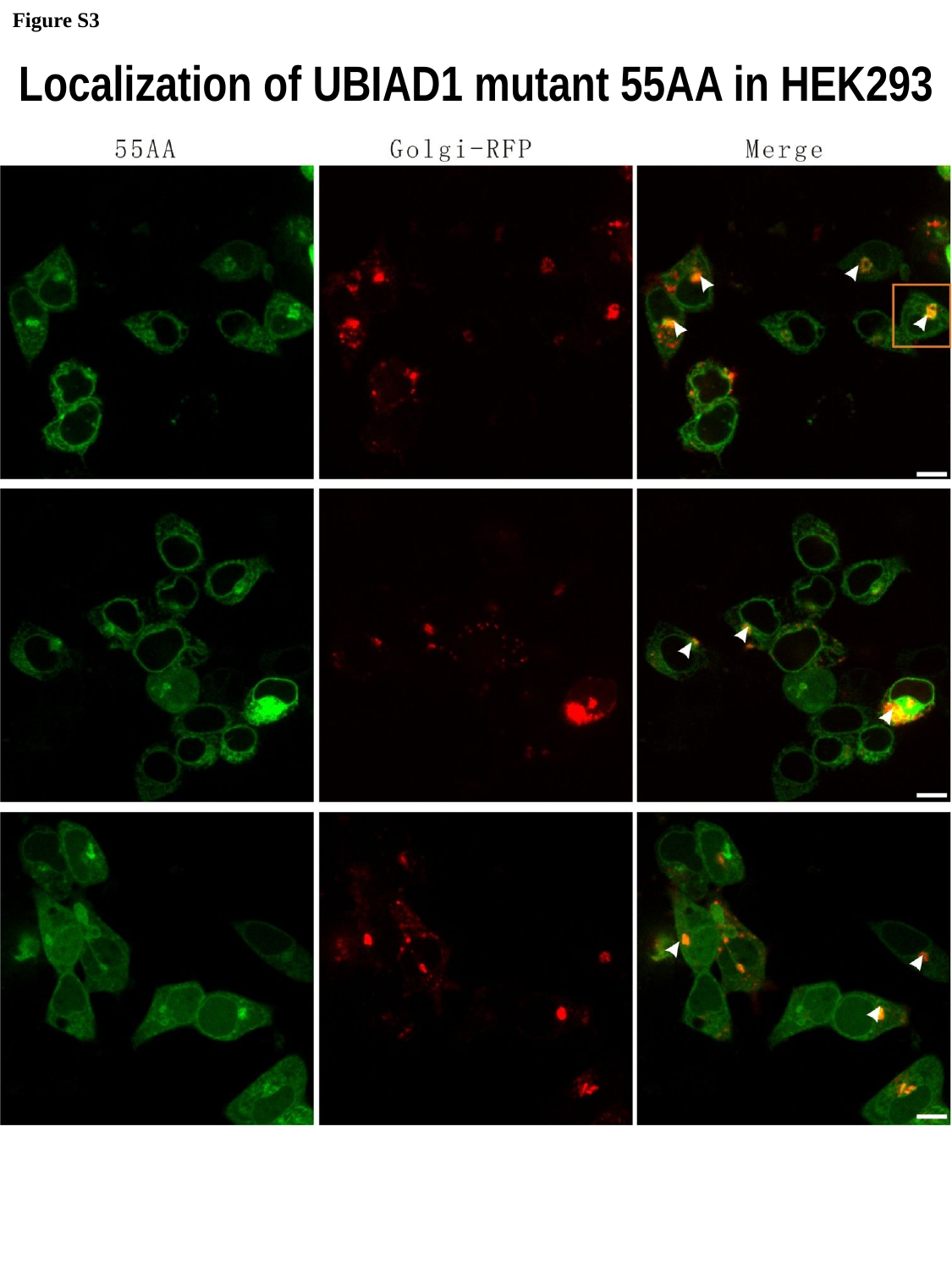

Figure S3
Localization of UBIAD1 mutant 55AA in HEK293

Supplement: Figure S3 — Localization of UBIAD1 mutant Δ55AA in HEK293 cells. Arrows point to the colocalization of UBIAD1-EGFP (Delta 55AA) and the Golgi. Bar represents 10 µm. Manders coefficients (M1 = 0.986, M2 = 0.988) were derived by using the IMAGE J software (using the framed cell as an example). (PPT) [file pone.0072015.s003.ppt]

## Slide 1
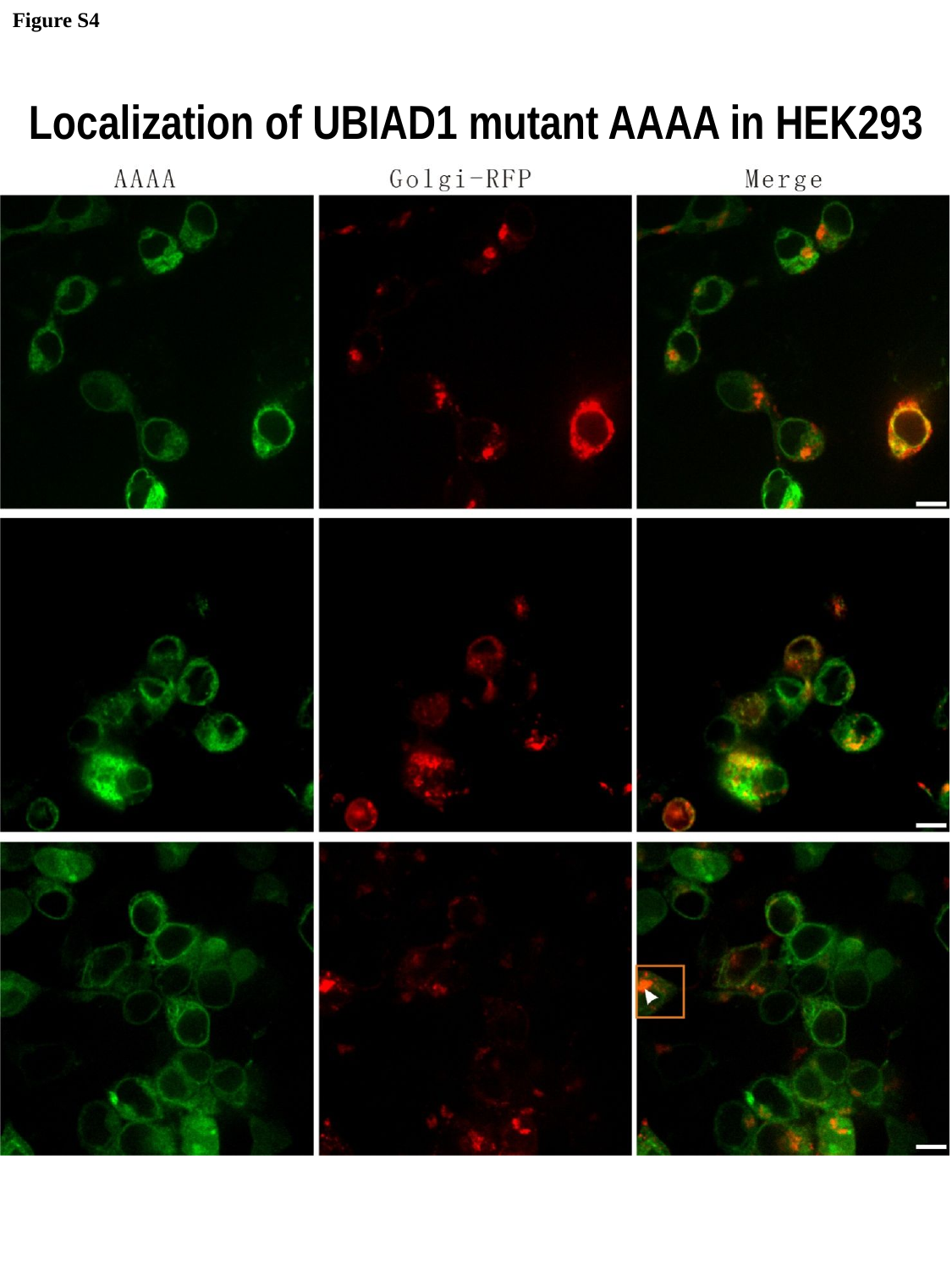

Figure S4
Localization of UBIAD1 mutant AAAA in HEK293

Supplement: Figure S4 — Localization of UBIAD1 mutant AAAA in HEK293 cells. Arrows point to the colocalization of UBIAD1-EGFP (AAAA) and the Golgi. Bar represents 10 µm. Manders coefficients (M1 = 0.969, M2 = 0.952) were derived by using the IMAGE J software (using the framed cell as an example). (PPT) [file pone.0072015.s004.ppt]

## Slide 1
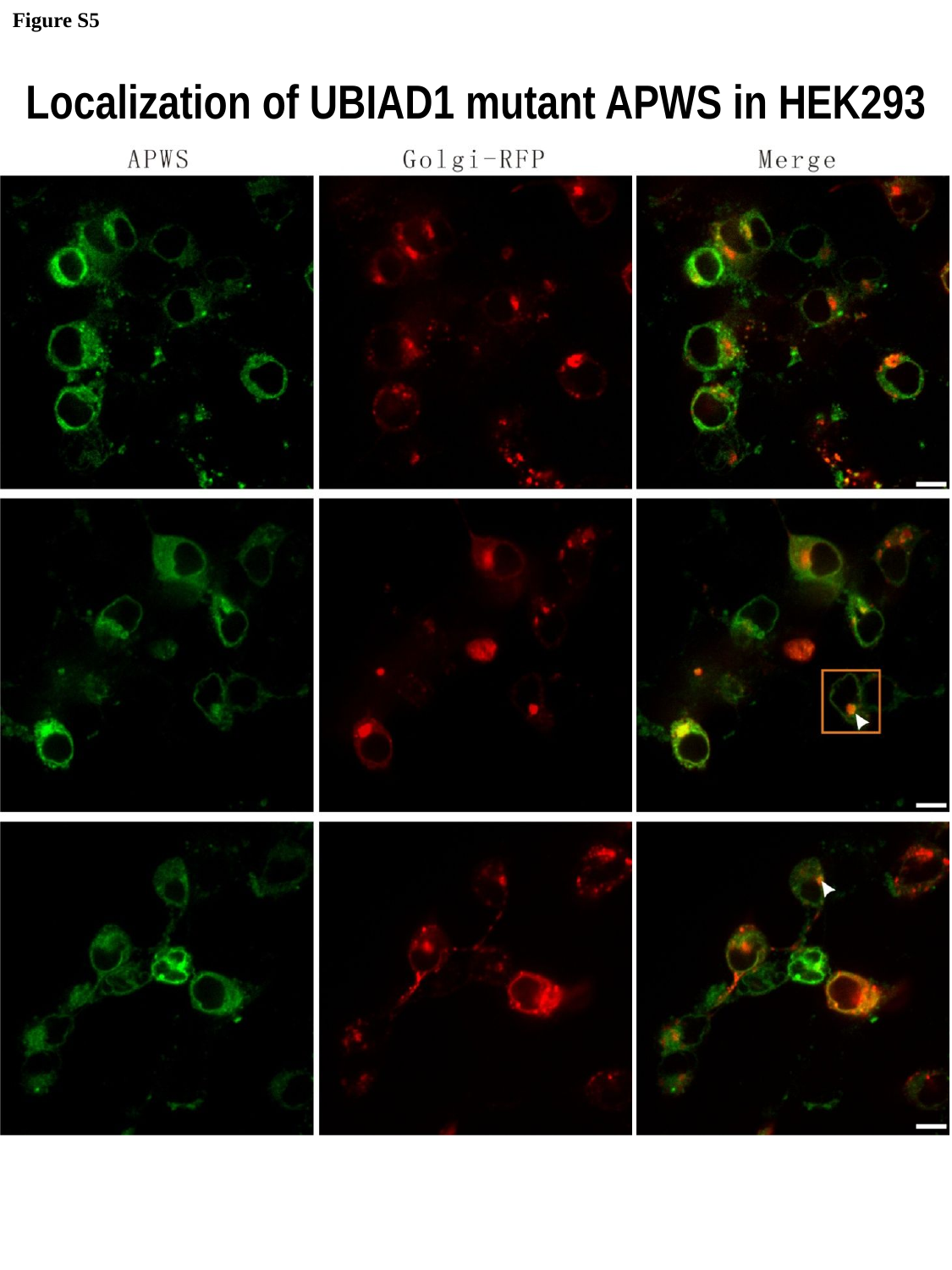

Figure S5
Localization of UBIAD1 mutant APWS in HEK293

Supplement: Figure S5 — Localization of UBIAD1 mutant APWS in HEK293 cells. Arrows point to the colocalization of UBIAD1-EGFP (APWS) and the Golgi. Bar represents 10 µm. Manders coefficients (M1 = 0.936, M2 = 0.985) were derived by using the IMAGE J software (using the framed cell as an example). (PPT) [file pone.0072015.s005.ppt]

## Slide 1
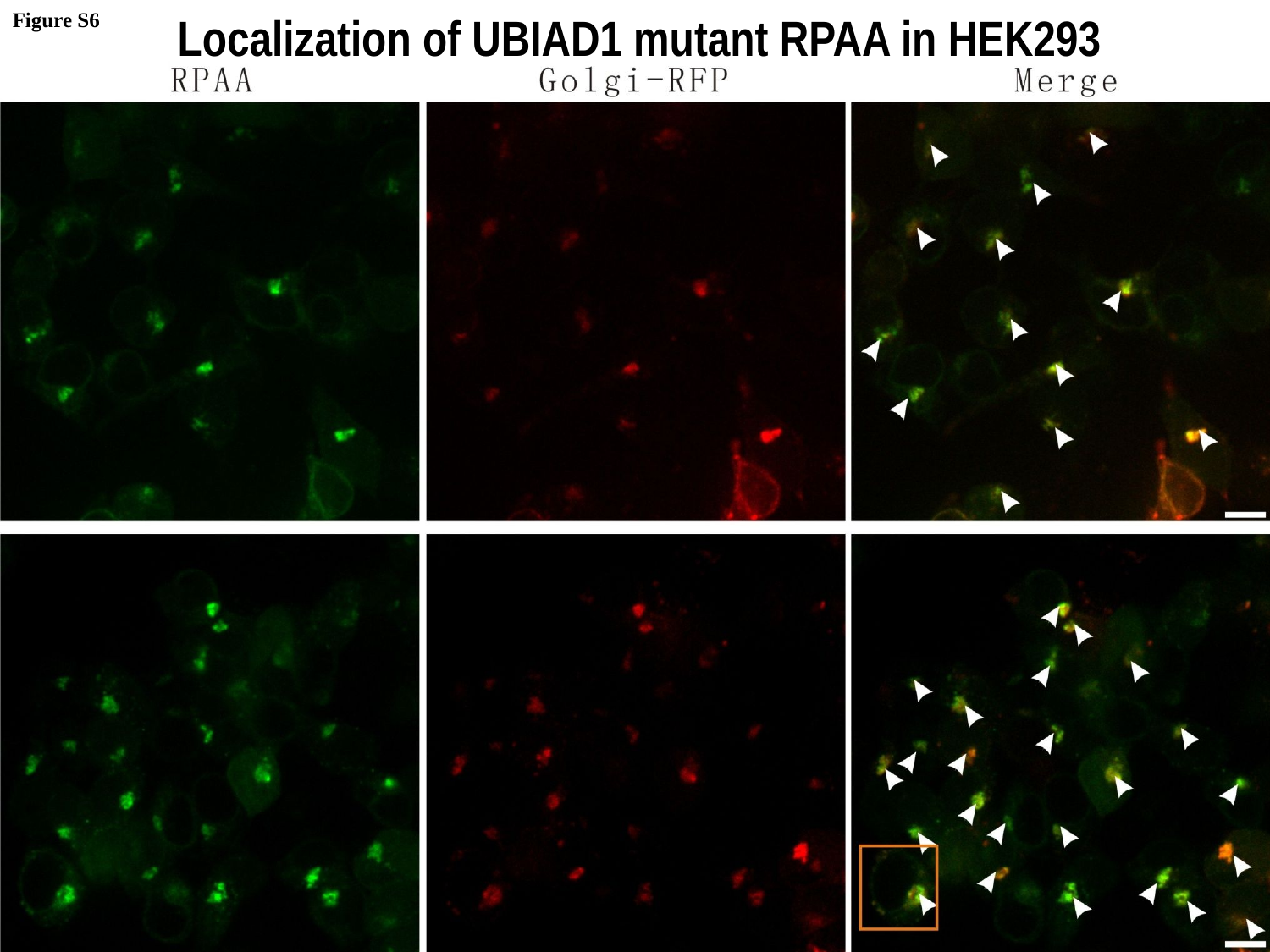

Figure S6
Localization of UBIAD1 mutant RPAA in HEK293

Supplement: Figure S6 — Localization of UBIAD1 mutant RPAA in HEK293 cells. Arrows point to the colocalization of UBIAD1-EGFP (RPAA) and the Golgi. Bar represents 10 µm. Manders Coefficients (M1 = 0.965, M2 = 0.711) were derived by using the IMAGE J software (using the framed cell as an example). (PPT) [file pone.0072015.s006.ppt]
